# Synthetic Optimization and MAPK Pathway Activation Anticancer Mechanism of Polyisoprenylated Cysteinyl Amide Inhibitors

**DOI:** 10.3390/cancers13225757

**Published:** 2021-11-17

**Authors:** Nada Tawfeeq, Yonghao Jin, Nazarius S. Lamango

**Affiliations:** College of Pharmacy and Pharmaceutical Sciences, Institute of Public Health, Florida A&M University, Tallahassee, FL 32307, USA; nada1.tawfeeq@famu.edu (N.T.); yonghao.jin@famu.edu (Y.J.)

**Keywords:** PCAIs, MAPK, KRAS, MEK1/2, ERK1/2, p90RSK

## Abstract

**Simple Summary:**

RAS G-protein genes are frequently mutated and drive the progression of about 30% of human cancers. Polyisoprenylated cysteinyl amide inhibitors (PCAIs) offer a novel approach to address the decades-long anti-RAS drug development challenge. This manuscript reports on the continuous development of the PCAIs and their anticancer molecular mechanisms that involve strong activation of MAP kinase pathway enzymes.

**Abstract:**

Abnormalities of the MAPK pathway play vital roles in cancer initiation and progression. RAS GTPases that are key upstream mediators of the pathway are mutated in 30% of human cancers. Polyisoprenylated cysteinyl amide inhibitors (PCAIs) were designed as potential targeted therapies against the RAS-driven cancers. The current study reports on the optimization of the PCAIs and the determination of their mechanisms of action in KRAS-mutant cancer cells. They display ClogP values ranging from 3.01 to 6.35, suppressing the viabilities of KRAS-mutant MDA-MB-231, A549, MIA PaCa-2, and NCI-H1299 cells in 2D and 3D cultures with EC_50_ values of 2.2 to 6.8, 2.2 to 7.6, 2.3 to 6.5 and 5.0 to 14 µM, respectively. When A549 cells were treated with the PCAIs, NSL-YHJ-2-27, for 48 h, no significant difference was observed in the levels of total or phosphorylated B- and C-Raf proteins. However, at 5 µM, it stimulated the phosphorylation of MEK1/2, ERK1/2, and p90RSK by 84%, 59%, and 160%, respectively, relative to controls. A non-farnesylated analog, NSL-YHJ-2-62, did not elicit similar effects. These data reveal that effects on the RAS-MAPK signaling axis most likely contribute to the anticancer effects of the PCAIs, possibly through the proapoptotic isoforms of p90RSK. The PCAIs may thus have the potential to serve the unmet therapeutic needs of patients with aberrant hyperactive G-protein signaling.

## 1. Introduction

RAS small GTPases are key upstream mediators of the mitogen-activated protein kinase (MAPK) pathway, a key signaling pathway that regulates normal cell growth, differentiation, and survival [1]. Activation of this pathway is initiated by the binding of the epidermal growth factor (EGF) to its cell surface receptor [2]. Activation of the intracellular kinase domains of the receptors results in an intracellular chain of events that involves the exchange of GDP for GTP on RAS small GTPases [3]. The activated GTP-bound RAS proteins are deactivated through their intrinsic GTPase activity [3]. GTPase-activating proteins (GAPs) are essential factors for the hydrolysis of GTP to GDP and inorganic phosphate [4]. In fact, some cases of cancer involve mutational loss-of-function of GAPs that renders otherwise normal RAS proteins abnormally hyperactive, thereby also acting as cancer drivers [5]. Activated RAS proteins recruit rapidly accelerated fibrosarcoma (RAF) to the membrane for activation through phosphorylation [2]. Activated RAF phosphorylates and activates MEK1/2, which then activates ERK1/2 through phosphorylation [2]. Active ERK1/2 catalyzes the phosphorylation of a large number of substrates both in the cytoplasm and nucleus [6]. Some of ERK1/2 substrates are the p90 ribosomal S6 kinases (p90RSKs), which consist of four isoforms (p90RSK1-4) [7,8]. 

Overexpression of the growth factors constitutes causative and progressive factors in various neoplasms [9]. In others, overexpression of the receptors and/or gain-in-function mutations of the intracellular enzyme domains are the principal drivers [10,11]. While activating RAS mutations drive progression in many cases, as stated above, mutant ineffective GAP proteins are the drivers in numerous other cases such as in breast cancer [5]. Abnormalities of the RAS-RAF-MEK-ERK signal transduction cascade downstream of the receptors play vital roles in the initiation and progression of other cancers. For example, RAS G-proteins are frequently mutated oncogenes in a large proportion of human cancers, accounting for up to 30% of cases. KRAS is the most frequently mutated of the RAS isoforms, accounting for up to 86% of RAS mutant cancer cases [12]. Mutant KRAS is prevalent in lung, colon, and pancreatic cancers, making it one of the most aggressive and deadliest cancers in the US [1,13,14]. Mutant RAS-driven cancers are aggressive thanks to their inability to hydrolyze GTP [15]. While thus being constitutively active, their high affinity for GTP means that it has so far been impossible to develop chemical agents that can displace them [3,16], thus rendering the mutant RAS oncoproteins virtually impossible to drug. Another major challenge to drugging RAS was met when prenylation inhibitors that were designed to block the essential post-translational modifications were found to be unacceptably toxic [17]. 

The polyisoprenylated cysteinyl amide inhibitors offer a novel avenue to address the decades-long oncogenic RAS conundrum [18]. The PCAIs’ design strategy requires a rather hydrophobic polyisoprenyl cysteinyl amide pharmacophore that needs to be counterbalanced to attain therapeutically suitable aqueous solubility. A side chain with at least one ionizable basic group contributes to significant aqueous solubility in its salt form. However, the overall hydrophobicity of the molecules in our previous study [19] meant that improvements that diminish the overall hydrophobicity of the PCAIs are necessary. The synthetic optimization of the molecules to improve the aqueous solubilities is embodied in the series of PCAIs reported in the current study. We obtained analogs that are equally as potent against cancer cell viability as previous PCAIs but with significantly lower ClogP values. Furthermore, we show a possible mechanism with which the analogs activate MAPK pathway intermediates downstream of RAS proteins. 

## 2. Materials and Methods

### 2.1. General Procedures

Unless otherwise noted, all reagents from commercial sources were used without further purification. Reactions were monitored by thin-layer chromatography (TLC) using TLC plates precoated with TLC silica gel 60 F254 (Merck KGaA). Spots on TLC were visualized either directly with an ultraviolet light or after staining with *p*-anisaldehyde stain. Flash column chromatography was conducted on 40–63 µm silica gel. Unless otherwise stated, NMR spectra were obtained on a Varian Mercury 300 (300 MHz) in CDCl_3_. The reported chemical shifts for the ^1^H NMR spectra were recorded in parts per million (ppm) on the δ scale from an internal tetramethylsilane standard (0.0 ppm) and reported consecutively as position, multiplicity (s = singlet, d = doublet, t = triplet, q = quartet, dd = doublet of doublets, m = multiplet, and br = broad), coupling constant (J/Hz), relative integral, and assignment. High-resolution mass spectra (HRMS) were obtained on a Waters Acquity UPLC system equipped with a ThermoFisher QExactive mass spectrometer with electrospray ionization (ESI) operated in positive ionization mode. The purity of the final products was determined by HPLC.

### 2.2. Materials

All cell lines were purchased from American Type Culture Collection (ATCC, Manassas, VA, USA). MDA-MB-231, A549, and MIAPaCa-2 cells were cultured in high glucose Modified Eagle Medium (DMEM, Genesee Scientific, San Diego, CA, USA). NCI-H1299 cells were cultured in RPMI 1640 (Genesee Scientific, San Diego, CA, USA). All media were supplemented with 10% heat-inactivated fetal bovine serum (Genesee Scientific, San Diego, CA, USA), 100 U/mL penicillin, and 100 μg/mL streptomycin (Genesee Scientific, San Diego, CA, USA). The cultures were incubated at 37 °C in 5% CO_2_/95% humidified air. In all cases, treatment with experimental agents was done in basal medium supplemented with 5% heat-inactivated fetal bovine serum. Antibodies specific to B-Raf (Cat. #14814), Phospho-B-Raf (Ser445) (Cat. #2696), c-Raf (Cat. #53745), Phospho-c-Raf (Ser338) (Cat. #9427), MEK1/2 (Cat. #8727), Phospho-MEK1/2 (Ser217/221) (Cat. #9154), p44/42 MAPK (Erk1/2) (Cat. #4695), RSK1/RSK2/RSK3 (Cat. #9355), Phospho-p90RSK (Ser380) (Cat. #11989), GAPDH (HRP Conjugate) (Cat. #8884), α-Actinin (HRP Conjugate) (Cat. #12413), and anti-rabbit IgG, HRP-linked Antibody (Cat. #7074) were purchased from Cell Signaling Technology (Danvers, MA, USA).

### 2.3. Organic Synthesis of the PCAIs

**Scheme 1:**
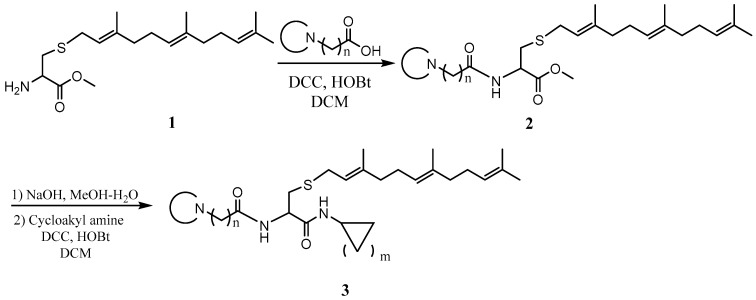
General procedure for the synthesis of the PCAIs.

General procedure for the synthesis of *L*-((cyclicamino)acyl)-S-(*trans, trans*-farnesyl)cysteine cycloalkyl amide and *L*-((cyclicamino)acyl)-S-(*all trans*-geranylgeranyl)cysteine cycloalkyl amide. To a solution of *L*-S-(*trans, trans*-farnesyl) cysteine methyl ester or *L*-S-(*all trans*-geranylgeranyl) cysteine methyl ester (1 eq), cyclicamino carboxylic acid (1 eq) and HOBt (1 eq) in CH_2_Cl_2_ (DCM, 0.1 M) at 0 °C was added N, N’-dicyclohexylcarbodiimide (DCC) (1 eq), and the resulting solution was stirred overnight. After filtration, the solution was washed with saturated aqueous NaHCO_3_ and dried over anhydrous Na_2_SO_4_. After filtering, the solvent was evaporated, and the residue was subjected to flash column separation (30% MeOH in ethyl acetate) to produce the methyl ester products as oils. To the methanol solution of the methyl ester (1 eq) at 0 °C was added aqueous NaOH solution (2 eq), and the resulting mixture was stirred at room temperature until no starting material was detected by TLC. The solution was acidified with 2N HCl aqueous solution and evaporated under vacuum. The residue was dissolved in DCM and filtered. The CH_2_Cl_2_ solution was cooled to 0 °C and treated with cycloalkyl amine (1eq), HOBt (1 eq), and DCC (1 eq). The mixture was stirred overnight and filtered. The solution was washed with saturated aqueous NaHCO_3_ and dried over anhydrous Na_2_SO_4_. After filtration, the solvent was evaporated and the residue separated on a flash chromatography column (50% MeOH in ethyl acetate) to afford the amide products as oils (Scheme 1). 

L-((4-methylpiperazinyl) acetyl)-S-(*trans, trans*-farnesyl) cysteine methyl ester (NSL-YHJ-2-22) ^1^HNMR (CDCl_3_, 300 MHz): δ 1.61 (s, 6H), 1.69 (s, 6H), 1.89-2.20 (m, 8H), 2.35 (s, 3H), 2.45–2.75 (m, 8H), 2.80–3.02 (m, 2H), 3.08 (s, 2H), 3.10–3.28 (m, 2H), 3.80 (s, 3H), 4.78 (m, 1H), 5.09 (m, 2H), 5.20 (m, 1H), 7.82 (br, 1H). HRMS (ESI) m/z: [M+H]^+^ calcd for C_26_H_46_N_3_O_3_S, 480.3255; found 480.3248. 

L-((4-methylpiperazinyl) acetyl)-S-(*trans, trans*-farnesyl) cysteine cyclopropyl amide (NSL-YHJ-2-48). ^1^HNMR: δ 0.52 (m, 2H), 0.76 (d, J = 4.0 Hz, 2H), 1.59 (s, 6H), 1.67 (s, 6H), 1.80–2.20 (m, 8H), 2.36 (s, 3H), 2.45–2.68 (m, 9H), 2.68–2.90 (m, 2H), 3.05 (s, 2H), 4.38 (m, 1H), 5.05 (m, 2H), 5.25 (m, 1H), 6.57 (br, 1H), 7.82 (br, 1H). HRMS (ESI) m/z: [M+H]^+^ calcd for C_28_H_49_N_4_O_2_S, 505.3571; found 505.3562. 

L-((4-methylpiperazinyl) acetyl)-S-(*trans, trans*-farnesyl) cysteine cyclobutyl amide (NSL-YHJ-2-47). ^1^HNMR: δ 1.57 (s, 6H), 1.68 (s, 6H), 1.80–2.15 (m, 10H), 2.31 (m, 7H), 2.40–2.68 (m, 8H), 2.82 (m, 2H), 3.03 (s, 2H), 3.18 (m, 2H), 4.17 (m, 2H), 5.08 (m, 2H), 5.22 (m, 1H), 6.60 (br, 1H), 7.82 (br, 1H). HRMS (ESI) m/z: [M+H]^+^ calcd for C_29_H_51_N_4_O_2_S, 518.3727; found 518.3718. 

L-((4-methylpiperazinyl) acetyl)-S-(*trans, trans*-farnesyl) cysteine cyclopentyl amide (NSL-YHJ-2-46). ^1^HNMR: δ 1.30–1.77 (m, 8H), 1.59 (s, 6H), 1.67 (s, 6H), 1.80–2.15 (m, 8H), 2.31 (s, 3H), 2.40–2.68 (m, 8H), 2.68–2.90 (m, 2H), 3.03 (s, 2H), 3.10 (m, 2H), 4.17 (m, 1H), 4.40 (m, 1H), 5.05 (m, 2H), 5.22 (m, 1H), 6.40 (br, 1H), 7.82 (br, 1H). HRMS (ESI) m/z: [M+H]^+^ calcd for C_30_H_53_N_4_O_2_S, 533.3884; found 533.5876. 

L-((4-methylpiperazinyl) acetyl)-S-(*trans, trans*-farnesyl) cysteine cyclohexyl amide (NSL-YHJ-2-45). ^1^HNMR: δ 1.10–1.43 (m, 10H), 1.59(s, 6H) 1.67 (s, 6H), 1.67 (s, 6H), 1.80–2.15 (m, 8H), 2.31 (s, 3H), 2.40–2.68 (m, 8H), 2.68–2.90 (m, 2H), 3.04 (s, 2H), 3.10 (m, 2H), 3.78 (m, 1H), 4.20 (m, 1H), 5.05 (m, 2H), 5.22 (m, 1H), 6.30 (br, 1H), 7.82 (br, 1H). HRMS (ESI) m/z: [M+H]^+^ calcd for C_31_H_55_N_4_O_2_S, 547.4040; found 547.4031. 

L-((4-methylpiperazinyl) acetyl)-S-(*trans, trans*-farnesyl) cysteine cycloheptyl amide (NSL-YHJ-2-44). ^1^HNMR: δ 1.16–1.70 (m, 12H), 1.59 (s, 6H), 1.67 (s, 6H), 1.80–2.15 (m, 8H), 2.33 (s, 3H), 2.40–2.68 (m, 8H), 2.68–2.90 (m, 2H), 3.04 (s, 2H), 3.19 (m, 2H), 3.90 (m, 1H), 4.40 (m, 1H), 5.08 (m, 2H), 5.24 (m, 1H), 6.38 (br, 1H), 7.83 (br, 1H). HRMS (ESI) m/z: [M+H]^+^ calcd for C_32_H_57_N_4_O_2_S, 561.4197; found 561.4188. 

L-((4-methylpiperazinyl) acetyl)-S-(*trans, trans*-farnesyl) cysteine cyclooctyl amide (NSL-YHJ-2-27). ^1^HNMR: δ 1.16–1.70 (m,14H), 1.59 (s, 6H), 1.67 (s, 6H), 1.80–2.15 (m, 8H), 2.33 (s, 3H), 2.40–2.68 (m, 8H), 2.68–2.90 (m, 2H), 3.04 (s, 2H), 3.19 (m, 2H), 3.90 (m, 1H), 4.40 (m, 1H), 5.08 (m, 2H), 5.24 (m, 1H), 6.38 (br, 1H), 7.83 (br, 1H). HRMS (ESI) m/z: [M+H]^+^ calcd for C_33_H_59_N_4_O_2_S, 575.4353; found 575.4346. 

L-((4-methylpiperazinyl) propanoyl)-S-(*trans, trans*-farnesyl) cysteine cyclooctyl amide (NSL-YHJ-2-35). ^1^HNMR: δ 1.16-1.70 (m, 14H), 1.59 (s,6H), 1.67 (s, 6H), 1.80–2.15 (m, 8H), 2.33 (s, 3H), 2.40–2.68 (m, 8H), 2.68–2.90 (m, 2H), 3.04 (s, 2H), 3.19 (m, 2H), 3.90 (m, 1H), 4.40 (m, 1H), 5.08 (m, 2H), 5.24 (m, 1H), 6.38 (br, 1H), 7.83 (br, 1H). HRMS (ESI) m/z: [M+H]^+^ calcd for C_34_H_61_N_4_O_2_S, 589.4500; found 589.4496. 

L-((4-methylpiperazinyl) butanoyl)-S-(*trans, trans*-farnesyl) cysteine cyclooctyl amide (NSL-YHJ-2-37). ^1^H NMR: δ 1.20–1.90 (m, 28H), 1.92–2.15 (m, 8H), 2.24 (t, 7.5Hz, 2H), 2.32 (s, 2H), 2.38 (t, 7.5Hz, 2H), 2.51 (br, 8H), 2.68 (dd, 14.1, 7.5 Hz, 1H), 2.87 (dd, 14.1, 5.4 Hz, 1H), 3.24 (m, 1H), 3.96 (m, 1H), 4.39 (m, 1H), 5.07 (m, 2H), 5.25 (m, 1H), 6.33 (br, 1H), 6.42 (m, 1H). HRMS (ESI) m/z: [M+H]^+^ calcd for C_35_H_63_N_4_O_2_S, 603.4666; found 603.4658. 

L-((4-methylpiperazinyl) pentanoyl)-S-(*trans, trans*-farnesyl) cysteine cyclooctyl amide (NSL-YHJ-2-40). ^1^H NMR: δ 1.20–1.90 (m, 30H), 1.92-2.15 (m, 8H), 2.24 (t, 7.5Hz, 2H), 2.32 (s, 2H), 2.38 (t, 7.5Hz, 2H), 2.51 (br, 8H), 2.68 (dd, 11.1, 7.5 Hz, 1H), 2.87 (dd, 9.0, 5.4 Hz, 1H), 3.24 (m, 1H), 3.95 (m, 1H), 4.39 (m, 1H), 5.08 (m, 2H), 5.26 (m, 1H), 6.33 (br, 1H), 6.42 (m, 1H). HRMS (ESI) m/z: [M+H]^+^ calcd for C_36_H_65_N_4_O_2_S, 617.4823; found 617.4815. 

L-((4-methylpiperazinyl) hexanoyl)-S-(*trans, trans*-farnesyl) cysteine cyclooctyl amide (NSL-BA-056). ^1^H NMR: δ 0.76–1.02 (m, 2H), 1.12–1.31 (m,2H), 1.37–1.83 (m, 32H), 2.02 (dd, J ¼ 25.3, 6.5 Hz, 6H), 2.21 (d,J ¼ 23.8 Hz, 2H), 2.83 (d, J ¼ 39.2 Hz, 5H), 3.04e3.27 (m, 4H), 3.66 (s,6H), 3.92 (s, 1H), 4.42 (s, 1H), 5.08 (d, J ¼ 6.6 Hz, 2H), 5.24 (s, 2H),6.55 (d, J ¼ 7.4 Hz, 1H), 6.99 (s, 1H). 

L-((4-methylpiperazinyl) acetyl)-S-(*all trans*-geranylgeranyl) cysteine methyl ester (NSL-YHJ-2-89-1). ^1^H NMR: δ 1.60 (s, 9H), 1.67 (s, 6H), 1.80–2.20 (m, 12H), 2.32 (s, 3H), 2.40–2.75 (br, 8H), 2.90 (m, 2H), 3.06 (s, 3H), 3.17 (m, 2H), 3.76 (s, 3H), 4.80 (m, 1H), 5.10 (m, 3H), 5.20 (m, 1H), 7.84 (br, 1H)). HRMS (ESI) m/z: [M+H]^+^ calcd for C_31_H_54_N_3_O_3_S, 643.4979; found 643.4966. 

L-((4-methylpiperazinyl) acetyl)-S-(*all trans*-geranylgeranyl) cysteine cyclooctyl amide (NSL-YHJ-2-GG). ^1^H NMR: δ 1.20–1.80 (m, 29H), 1.85–2.15 (br,12H), 2.30 (s, 3H), 2.30–2.65 (br, 8H), 2.80 (m, 2H), 3.00 (s, 2H), 3.20 (m, 2H), 3.96 (m, 1H), 4.40 (m, 1H), 5.06 (m, 2H), 5.25 (m, 1H), 6.38 (br, 1H), 7.82 (br, 1H). HRMS (ESI) m/z: [M+H]^+^ calcd for C_38_H_67_N_4_O_2_S, 548.3881; found 548.3889. 

L-S-(*trans, trans*-farnesyl) cysteine cyclooctyl amide (NSL-YHJ-096). *L-*S-*trans, trans*-Farnesyl cysteine methyl ester (1 eq) was dissolved in MeOH and treated with an aqueous solution of NaOH (2 eq). After complete ester hydrolysis as observed on TLC, the solution was treated with aqueous HCl (1 eq) and evaporated. The residue was dissolved in water and cooled to 0 °C. Fmoc-Cl (1.1 eq) in THF and 1N aqueous NaOH (1.1 eq) were added alternately. The mixture was stirred for 2 h and then acidified to pH 2 with 2N HCl aqueous solution. After evaporation, the residue was dissolved in ethyl acetate and washed with water. The solution was dried over Na_2_SO_4_ and filtered. After evaporation, the residue was subjected to flash column chromatography (50% ethyl acetate in hexane) to produce the product, Fmoc- *L-*S-(*trans, trans*-farnesyl) cysteine as an oil. To the DCM solution of Fmoc-S-(*trans, trans*-farnesyl) cysteine was added cyclooctyl amine (1eq) and HOBt (1 eq). The solution was cooled to 0 °C and treated with DCC (1 eq). The mixture was stirred overnight and filtered. The solution was washed with saturated aqueous NaHCO_3_ and dried over anhydrous Na_2_SO_4_. After filtration, the solvent was evaporated, and the residue was subjected to flash column separation (20% ethyl acetate in hexane) to produce the amide product as an oil. Pyrrolidine (20 eq) was added to the cysteine amide solution. After 30 min, the solution was evaporated under vacuum and the residue was purified by flash column (50% MeOH in ethyl acetate) to produce the product as an oil. ^1^HNMR: δ 1.16–1.70 (m, 26H), 1.90–2.20 (m, 8H), 2.45–3.80 (m, 5H), 3.90–4.10 (m, 1H), 5.10 (m, 2H), 5.30 (m, 1H), 7.25–7.40 (br, 1H). HRMS (ESI) m/z: [M+H]^+^ calcd for C_26_H_47_N_2_OS, 435.3404; found 435.3405. 

L-(3-Methoxycarbonylpropionyl)-S-(*trans, trans*-farnesyl) cysteine cyclooctyl amide (NSL-YHJ-2-84). To the solution of L-S-(*trans, trans*-farnesyl) cysteine cyclooctyl amide (1 eq), succinic acid monomethyl ester (1 eq), and HOBt (1 eq) in CH_2_Cl_2_ (DCM) (0.1 M) at 0 °C was added DCC (1 eq), and the resulting solution was stirred overnight. After filtration, the solution was washed with saturated aqueous NaHCO_3_ and dried over anhydrous Na_2_SO_4_. After filtration, the solvent was evaporated, and the residue was subjected to flash column separation (50% ethyl acetate in hexane) to produce the methyl ester product as an oil.^1^H NMR: δ 1.40–1.85 (m, 30H), 1.85–2.15 (m,8H), 2.49 (t, 5.5 Hz, 2H), 2.60(dd, 12.0, 6.9 Hz, 2H), 2.75 (m, 2H), 3.00 (dd, 13.8, 5.1Hz, 1H), 3.22 (d, 7.5 Hz, 2H), 3.68 (s, 3H), 3.96 (m, 1H), 4.47 (m, 1H), 5.08 (m, 2H), 5.24 (m, 1H), 6.52 (br, 2H).HRMS (ESI) m/z: [M+H]^+^ calcd for C_31_H_53_N_2_O_4_S, 549.3721; found 549.3718. 

L-(3-Hydroxycarbonylpropionyl)-S-(*trans, trans*-farnesyl) cysteine cyclooctyl amide (NSL-YHJ-3-36). To the methanol solution of L-(3-Methoxycarbonylpropionyl)-S-(*trans, trans*-farnesyl) cysteine cyclooctyl amide (1 eq) at 0 °C was added aqueous NaOH solution (2 eq), and the resulting mixture was stirred at room temperature until no starting material was detected on TLC plates. The solution was acidified to pH 2 with 2N HCl aqueous solution and extracted 3 times with ethyl acetate. The combined organic layer was dried over anhydrous Na_2_SO_4_. After filtration, the solvent was evaporated, and the residue was subjected to flash column separation (ethyl acetate) to produce the acid product as an oil. ^1^H NMR: δ 1.40–1.85 (m, 30H), 1.85–2.15 (m,8H), 2.55 (m, 2H), 2.70(m, 2H), 2.75 (m, 3H), 2.72 (m, 1H), 3.00 (m, 2H), 3.94 (m, 1H), 4.45 (m, 1H), 5.08 (m, 2H), 5.22 (m, 1H), 6.55 (br, 1H), 7.50 (br, 1H). HRMS (ESI) m/z: [M+H]^+^ calcd for C_30_H_51_N_2_O_4_S, 535.3570; found 535.3561.

L-((4-Methylpiperazinyl) acetyl)-S-methyl cysteine cyclooctyl amide (NSL-YHJ-2-62)). To a mixture of N-(*tert*-butoxycarbonyl)-L-cysteine methyl ester (1 eq) and K_2_CO_3_ (1 eq) in DMF at 0 °C was added MeI (1.1 eq). After stirring for 2 h, the mixture was poured into water and extracted 3 times with ethyl acetate. The combined ethyl acetate extracts were washed with water and dried over anhydrous Na_2_SO_4_. After filtration, the solvent was evaporated, and the residue was subjected to flash column separation (10% ethyl acetate in hexane) to produce the S-methylated product as an oil. To the methanol solution of Boc-S-methyl cysteine methyl ester (1 eq) at 0 °C was added aqueous KOH (2 eq), and the resulting mixture was stirred at room temperature until no starting material was detected on TLC. The solution was acidified to pH 2 with 2N HCl aqueous solution and evaporated under vacuum. The residue was dissolved in DCM and filtered. To the DCM solution was added cyclooctyl amine (1 eq) and HOBt (1 eq). The solution was cooled to 0 °C and treated with DCC (1 eq). The mixture was stirred overnight, filtered, and washed with saturated aqueous solution of NaHCO_3_ and dried over anhydrous Na_2_SO_4_. After filtration, the solvent was evaporated, and the residue subjected to flash column separation (20% ethyl acetate in hexane) to afford the amide product as an oil. The amide intermediate was treated with 3N HCl in MeOH until no starting material was detected on TLC. After evaporation of MeOH, the solid residue was dissolved in DCM and cooled to 0 °C. To the solution were added triethylamine (2.2 eq) and chloroacetyl chloride (1.1 eq). After 2 h, the reaction was quenched with water and washed with 2N HCl aqueous solution and saturated NaHCO_3_. The DCM solution was dried over anhydrous Na_2_SO_4_ and filtered; the solvent was evaporated under reduced pressure, and the residue was subjected to flash column separation (20% ethyl acetate in hexane) to produce the amide product as an oil. The intermediate was dissolved in toluene and 4-methylpiperazine (2 eq) was added. To the toluene solution was added K_2_CO_3_, and the resulting mixture was refluxed for 2 h. After cooling down, the reaction mixture was filtered, and the solvent removed under reduced pressure. The residue was subject to flash column separation (50% MeOH in ethyl acetate) to produce the amide product as an oil. ^1^HNMR: δ 1.16–1.70 (m, 14H), 2.17 (s, 3H), 2.29 (s, 3H), 2.40–2.68 (br, 8H), 2.68–3.0 (m, 2H), 3.03 (s, 2H), 3.95 (m, 1H), 4.05 (m, 1H), 6.38 (br, 1H), 7.88 (br, 1H). HRMS (ESI) m/z: [M+H]^+^ calcd for C_19_H_37_N_4_O_2_S, 385.2632; found 385.2618. 

L-6-Bromohexanoyl-alanine cyclooctyl amide (NSL-YHJ-2-111-1) ^1^H NMR: δ 1.34 (d, 7.2 Hz, 3H), 1.38–1.72 (m, 16H), 1.72–1.93 (m, 4H), 2.21 (t, 7.5Hz, 2H), 3.40 (t, 6.6 Hz), 3.94 (m, 1H), 4.42 (m, 1H), 6.22 (br, 2H). HRMS (ESI) m/z: [M+H]^+^ calcd for C_17_H_32_BrN_2_O_2_, 375.1642; found 375.1635. 

L-(6-Pyrrolidinylhexanoyl)-alanine cyclooctyl amide (NSL-YHJ-2-31) ^1^H NMR: δ 1.32 (m, 2H), 1.33 (d, 7.5 Hz, 3H), 1.40–1.85 (m, 22H), 2.18 (t, 7.5 Hz, 2H), 2.4 (m, 6H), 3.90 (m, 1H), 4.42 (m, 1H), 6.30 (br, 1H), 6.43 (br, 1H). HRMS (ESI) m/z: [M+H]^+^ calcd for C_21_H_40_N_3_O_2_, 366.3115; found 366.3103. 

L-((4-Methylpiperazinyl) acetyl)-alanine cyclooctyl amide (NSL-YHJ-2-56). ^1^H NMR: δ 1.35 (d, 7.5 Hz, 3H), 1.40–1.85 (m, 14H), 2.28 (s, 3H), 2.35–2.62 (br, 8H), 2.99 (s, 2H), 3.92 (m, 1H), 4.41 (m, 1H), 6.38 (br, 1H), 7.63 (br, 1H). HRMS (ESI) m/z: [M+H]^+^ calcd for C_18_H_35_N_4_O_2_, 339.2755; found 339.2749. 

### 2.4. Determination of PCAIs Effects on Cell Viability

To determine the effects of the PCAIs on cell viability, cells were seeded at a density of 1.0 × 10^4^ cells per well in 96-well tissue culture plates (Genesee Scientific, San Diego, CA, USA) and allowed to attach overnight at 37 °C in 5% CO_2_/95% humidified air. The cells were then treated with varying concentrations of the PCAIs (0.5–50 µM). The PCAIs were dissolved in acetone (1% final acetone concentrations in the wells). Control cells were treated with 1% acetone in experimental media. Identical amounts of the compounds were used to treat the cells at 24 h for the 48 h exposure. Cell viability was determined after 48 h using the resazurin reduction assay, whereby resazurin (20 µL, 0.02%) was added to each well and then incubated at 37 °C in 5% CO_2_/95% humidified air for 1 to 3 h depending on the cell line. Fluorescence intensities were determined with excitation set at 475 nm and emission set at 580 nm using GloMax Explorer Microplate Reader (Promega, Madison, WI, USA). Cell viability was expressed as the percentage of the fluorescence in the treated cells relative to that of the controls. EC_50_ values were then obtained from nonlinear regression plots of fluorescence intensities against the concentrations of the respective agents. 

### 2.5. Effect of PCAIs on 3D Cancer Cell Spheroid Cultures

Cells were cultured in three-dimensional conditions in vitro and used to determine the effect of the PCAIs on 3D cells. To achieve this, cells were seeded at a density of 5 × 10^3^ per well in Nunclon™ Sphera™ 96-well, U-shaped-bottom microplates (Thermo Scientific, Waltham, MA, USA) and allowed to grow overnight at 37 °C in 5% CO_2_/95% humidified air. The formed compact spheroids were then treated with vehicle (1% acetone in culture media) or PCAIs (1–50 µM). Identical amounts of PCAIs were used to supplement the samples at 24 h for 48 h of exposure. To determine viabilities, spheroids were stained with acridine orange/ethidium bromide (AO/EO, 5 μg/mL) following 48 h exposure to the PCAIs. Fluorescent and brightfield images were captured using the Nikon Eclipse Ti inverted microscope (4× magnification) equipped with the Nikon DS Qi2 camera (Nikon Instruments Inc., Melville, NY, USA). Growth areas and the ratios of the fluorescent intensities of AO over EB for the respective PCAIs concentrations used were computed, and the data were graphed using GraphPad Prism version 8.0 for Windows (San Diego, CA, USA). 

### 2.6. Determination of the Mechanism of PCAIs-Induced Cell Death

The mode of cell death induced by the PCAIs was determined using the EB/AO and Annexin V/Propidium iodide flow cytometry approaches using A549 cells. Cells were cultured and treated once with NSL-YHJ-2-27 (0–10 µM). After 48 h, the cells were treated with AO/EB (10 mg/mL), incubated at room temperature for 10 min, and then imaged using the Nikon Eclipse Ti inverted microscope (10× magnification) equipped with the Nikon DS Qi2 camera (Nikon Instruments Inc. Melville, NY). For the Annexin V/PI staining, the annexin V-FITC apoptosis detection kit (MilliporeSigma, St. Louis, MO, USA) was used as per the manufacturer’s instructions. Briefly, cells were cultured and treated once with NSL-YHJ-2-27 (0–10 µM), and after 48 h, cells were washed with 1× PBS and harvested using 0.05% trypsin/EDTA. They were then washed with ice-cold 1× PBS and centrifuged. After washing, cells were resuspended in 1× binding buffer at a concentration of 10^6^ cells/mL. The cell suspension (200 µL) was added to the analysis tubes and stained with Annexin V-FITC and/or PI (1 µg/ml) and incubated in the dark at room temperature for 15 min. Flow cytometry was immediately performed on a Becton Dickinson FACSort flow cytometer with CellQuest software (Mansfield, MA, USA).

### 2.7. Effect of PCAIs on the Activation of MAPK Pathway Intermediates

A549 cells in complete medium were plated onto 60.8 cm^2^ tissue culture dishes (Genesee Scientific, San Diego, CA) at densities of 7 × 10^5^ cells/dish and then incubated for 24 h to allow the cells to adhere. Adherent cells were treated with 0–5 μM concentrations of PCAIs in experimental medium. After 24 h, equivalent amounts of PCAIs were used to supplement the treatments for the 48 h exposure. Cells were washed with PBS and then lysed with RIPA buffer (Genesee Scientific, San Diego, CA) supplemented with 0.1% *v*/*v* protease and phosphatase inhibitor cocktail (Cell Signaling Technology, Danvers, MA). The amount of protein in lysates was determined using the Quick Start™ Bradford protein assay (Bio-Rad, Hercules, CA, USA). Cell lysates containing equal amounts of protein (20–30 μg) were boiled in XT sample buffer with XT reducing agent (Bio-Rad, Hercules, CA, USA). The samples were separated by SDS-PAGE on 12% Criterion™ XT Bis-Tris protein gels and transferred onto Trans-Blot turbo midi 0.2 µm nitrocellulose membranes (Bio-Rad, Hercules, CA, USA). Membranes were blocked for 1 h at room temperature with OneBlock™ western-CL blocking buffer (Genesee Scientific, San Diego, CA, USA) and incubated overnight in blocking buffer containing rabbit monoclonal antibodies against the target proteins at 4 C. Membranes were then washed with 1X TBST and incubated with anti-rabbit IgG, HRP-linked antibody at room temperature for 2 h. Immunoreactive bands were then visualized using ProSignal^®^ Pico (Genesee Scientific, San Diego, CA, USA) or Radiance Plus (Azure Biosystems, Dublin, CA, USA) ECL reagents per manufacturers’ recommendations using the ChemiDoc XRS+ System (Bio-Rad, Hercules CA, USA). Protein levels as judged by the chemiluminescent intensities were quantified using Image Lab 6.0 (Bio-Rad, Hercules, CA, USA), normalized against the corresponding band intensities of either GAPDH or α-Actinin. The results were then plotted using GraphPad Prism version 8.0 for Windows (San Diego, CA, USA). 

### 2.8. Statistical Analysis

All cell viability results were expressed as the means of three independent experiments. The concentration–response curves were obtained by plotting the percent residual cell viabilities against the log of the inhibitor concentrations. Nonlinear regression plots were generated using GraphPad Prism version 8.0 for Windows (San Diego, CA, USA). From these, the concentrations that inhibited 50% of the cell viability were obtained. To determine statistical significance, the values of each treatment group were compared to the respective controls by One-Way ANOVA with Dunnett’s post-hoc test, and p values less than 0.05 were considered to be significant.

## 3. Results

### 3.1. Chemistry

**Scheme 2:**
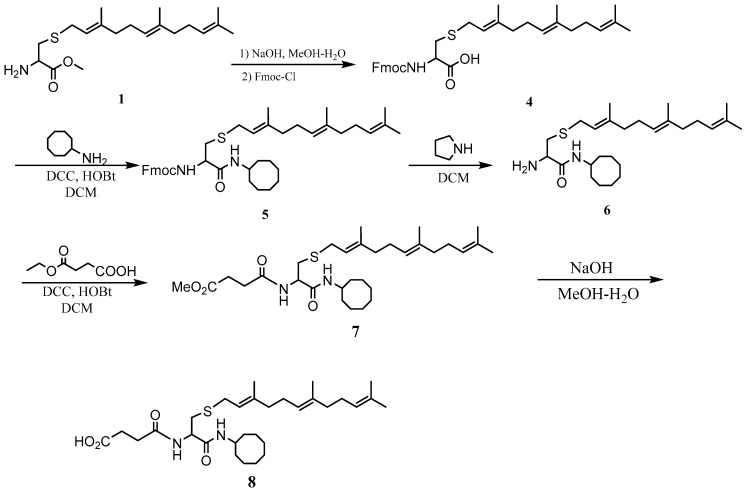
Synthesis of neutral and negatively charged PCAIs (NSL-YHJ-096 and NSL-YHJ-2-44).

**Scheme 3:**
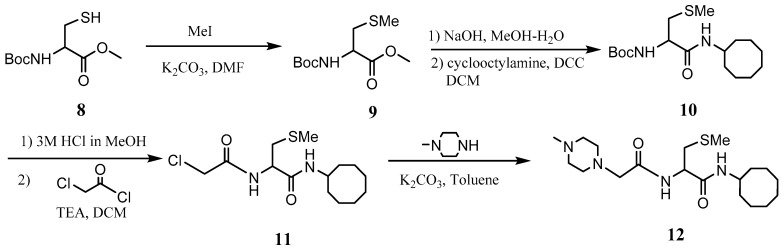
Synthesis of cysteine-based analogs lacking the polyisoprenyl moiety (NSL-YHJ-2-62).

**Scheme 4:**
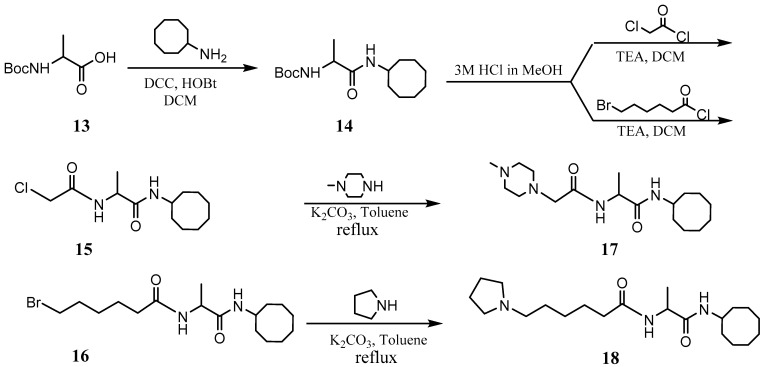
Synthesis of alanine-based analogs lacking the polyisoprenyl moiety (NSL-YHJ-2-31 and NSL-YHJ-2-56).

The PCAIs were synthesized according to Scheme 1 and Scheme 2. *L*-S-*trans, trans*-farnesyl cysteine methyl ester 1 was coupled with the respective 4-methylpiperazinyl carboxylic acids to produce the intermediates 2. The respective intermediates 2 were hydrolyzed under basic conditions, and the resulting carboxylic acids were coupled with cycloalkylamines to afford the final products 3. NSL-YHJ-096 and NSL-YHJ-2-44 were prepared according to Scheme 2. *L*-S-*trans, trans*-farnesyl cysteine methyl ester 1 was hydrolyzed, and the amine group was protected with Fmoc group to produce intermediate 4. The intermediate 4 was then coupled with cyclooctylamine to produce the amide 5. Fmoc deprotection in pyrrolidine gave NSL-YHJ-096, which was coupled with succinic acid monomethyl ester to produce NSL-YHJ-2-44. The control compound NSL-YHJ-2-62 was synthesized according to Scheme 3. *N-(tert*-butoxycarbonyl)-*L*-cysteine methyl ester 8 was S-methylated to produce S-methylated intermediate 9. The intermediate 9 was hydrolyzed and coupled with cyclooctylamine to produce the amide 10. The Boc protecting group of the cyclooctylamide 10 was removed under acidic conditions, and the resulting amine was treated with chloroacetyl chloride to produce intermediate 11. The intermediate 11 was treated with 1-methylpiperazine in toluene under reflux using anhydrous K_2_CO_3_ as base to yield the control compound NSL-YHJ-2-62. The alanine control compounds NSL-YHJ-2-31 and NSL-YHJ-2-56 were prepared similarly based on Scheme 4 using *N-(tert*-butoxycarbonyl)-*L*-alanine *13* as starting material. Boc-Ala-OH 13 was coupled with cyclooctyl amine to produce amide *14.* The Boc protecting group of the cyclooctylamide 14 was removed under acidic conditions, and the resulting amine was treated with chloroacetyl chloride or 6-bromohexanoyl chloride to produce intermediates 15 or 16. The intermediate 15 was treated with 1-methylpiperazine in toluene under reflux conditions using anhydrous K_2_CO_3_ as base to yield the control compound NSL-YHJ-2-31. Under the same conditions, the intermediate 16 was treated with pyrrolidine to produce the control compound NSL-YHJ-2-56. 

### 3.2. PCAIs Suppress Cancer Cell Viability

**Figure 1:**
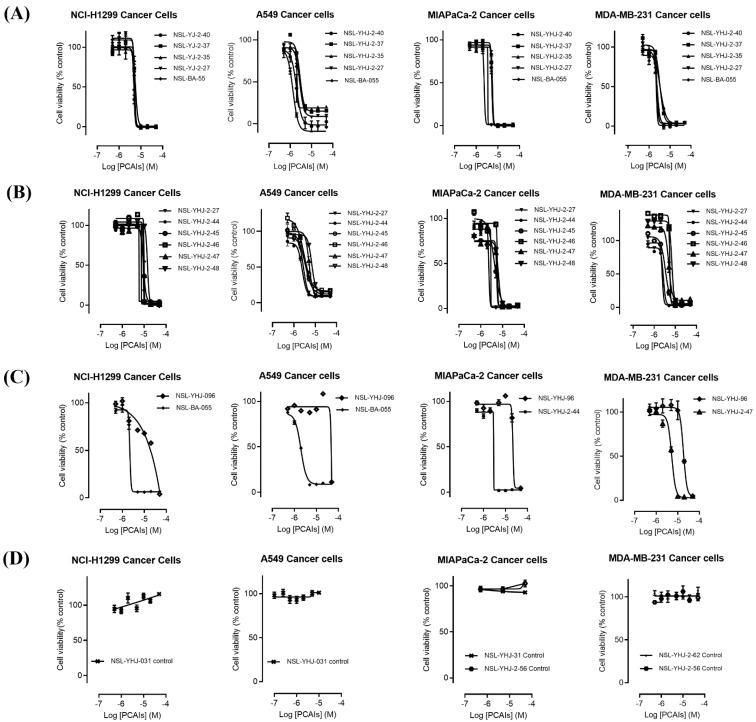
PCAIs analogs inhibit cancer cell viability. The effects of the PCAIs on the viability of human lung (A549 and NCI-H1299), pancreatic (MIA PaCa-2), and triple-negative breast (MDA-MB-231) cancer cells were determined following 48 h exposure to the respective PCAIs using the resazurin reduction assay. Each point represents the mean ± SEM, *n* = 3 relative to the control untreated cells. Concentration-response curves of (**A**) PCAIs with varying linker chain lengths, (**B**) PCAIs with varying cycloalkyl ring sizes, and (**C**) PCAIs with a free α-amino on the cysteine (NSL-YHJ-096) compared to some of the potent PCAIs analog; (**D**) analogs lacking the polyisoprenyl moiety showed no effects against cell viability.

**Figure 2:**
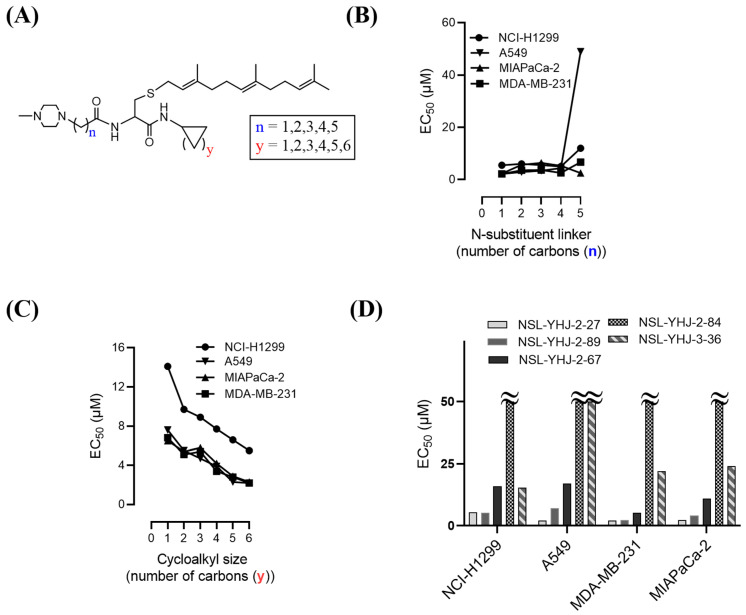
Structure–activity relationships. (**A**) PCAIs general structure showing areas of structural variation depicted by “n” and “y”; (**B**) effect on potency of PCAIs’ tether (linker/N-substituent chain) lengths; (**C**) effect on potency of the N-cycloalkyl ring size; and (**D**) comparative effectiveness of S-farnesylated (NSL-YHJ-2-27) versus S-geranylgeranylated (NSL-YHJ-2-89), pyrollidine-capped (NSL-YHJ-2-67) versus methylpiperidine-capped (NSL-YHJ-2-27), and ester-capped (NSL-YHJ-2-84) versus carboxylic acid-capped (NSL-YHJ-3-36) tethers.

The effect of the new PCAIs analogs on cancer cell viability was determined against MDA-MB-231 triple-negative breast cancer cells, A549 lung cancer cells, MIAPaCa-2 pancreatic cancer cells that harbor the mutant K-RAS oncogene, and NCI-H1299 lung cancer cells that have the mutant N-RAS oncogene. Treatment of cancer cell lines with PCAIs for 48 h resulted in significant concentration-dependent decreases in cell viability compared with untreated cells or cells treated with control compounds lacking the farnesyl group (Figure 1A,B). As shown in Table 1, the PCAIs suppressed the viabilities of MDA-MB-231, A549, MIA PaCa-2, and NCI-H1299 cells with 48 h EC_50_ values ranging from 2.2 to 6.8, 2.2 to 7.6, 2.3 to 6.5, and 5.0 to 14 µM, respectively. The PCAIs with the free α-amino (color-coded purple in Table 1) on the cysteine (NSL-YHJ-096) displayed a significant loss in potency with EC_50_ values ranging from 22 to over 50 µM against the different cell lines (Figure 1C). Reduction of the α-amino N-substituent size (color-coded blue in Table 1) decreased the hydrophobicity while maintaining the potency, with EC_50_ values of 5.5, 2.2, 2.2, and 2.3 µM for NSL-YHJ-2-27 with 2-carbon linker compared to 12, 49, 6.7, and 2.5 for NSL-BA-056 with 6-carbon linker against NCI-H1299, A549, MDA-MB-231, and MIA PaCa-2, respectively (Table 1, Figure 2A,B). Increasing the N-cycloalkyl ring size (color-coded red in Table 1) increased the log P values by over two-fold but resulted in an almost three-fold increase in potency. For example, the EC_50_ values of 5.5, 2.2, 2.2, and 2.3 µM for NSL-YHJ-2-27, which has the cyclooctyl ring is about three-fold more potent than NSL-YHJ-2-48 with the cyclopropyl ring with corresponding EC_50_ values of 14, 7.6, 6.8, and 6.5, respectively (Table 1 and Figure 2A,C). Analogs lacking the polyisoprenyl moiety (NSL-YHJ-2-56 and NSL-YHJ-2-62 vs. NSL-YHJ-2-27, and NSL-YHJ-31 vs. NSL-BA-055 color-coded orange in Table 1) used as control compounds had no effect on cell viability even at 50 µM (Figure 1D). This clearly demonstrates the importance of the farnesyl group to the PCAIs’ effectiveness. 

Use of pyrrolidine group instead of the methylpiperazine to cap the side chain linker decreased the potency by over 2.3- to 7.7-fold. For example, NSL-YHJ-2-67 with EC_50_ values of 16, 17, 5.2, and 11 µM for the different cell lines is less potent as compared to corresponding EC_50_ values of 5.5, 2.2, 2.2, and 2.3 µM, respectively, for NSL-YHJ-2-27 (Table 1). Use of the 20-carbon all trans-geranylgeranyl group as in NSL-YHJ-2-89 instead of the 15-carbon trans, trans-farnesyl group as in NSL-YHJ-2-27 did not result in significant changes in potency. Replacing a positively charged side chain with one that is either neutral or negatively charged at physiological pH as in compounds NSL-YHJ-2-84 and NSL-YHJ-3-36 results in significant losses in potency (Table 1, Figure 2D).

### 3.3. PCAIs Induce the Degeneration of 3D Spheroids

**Figure 3:**
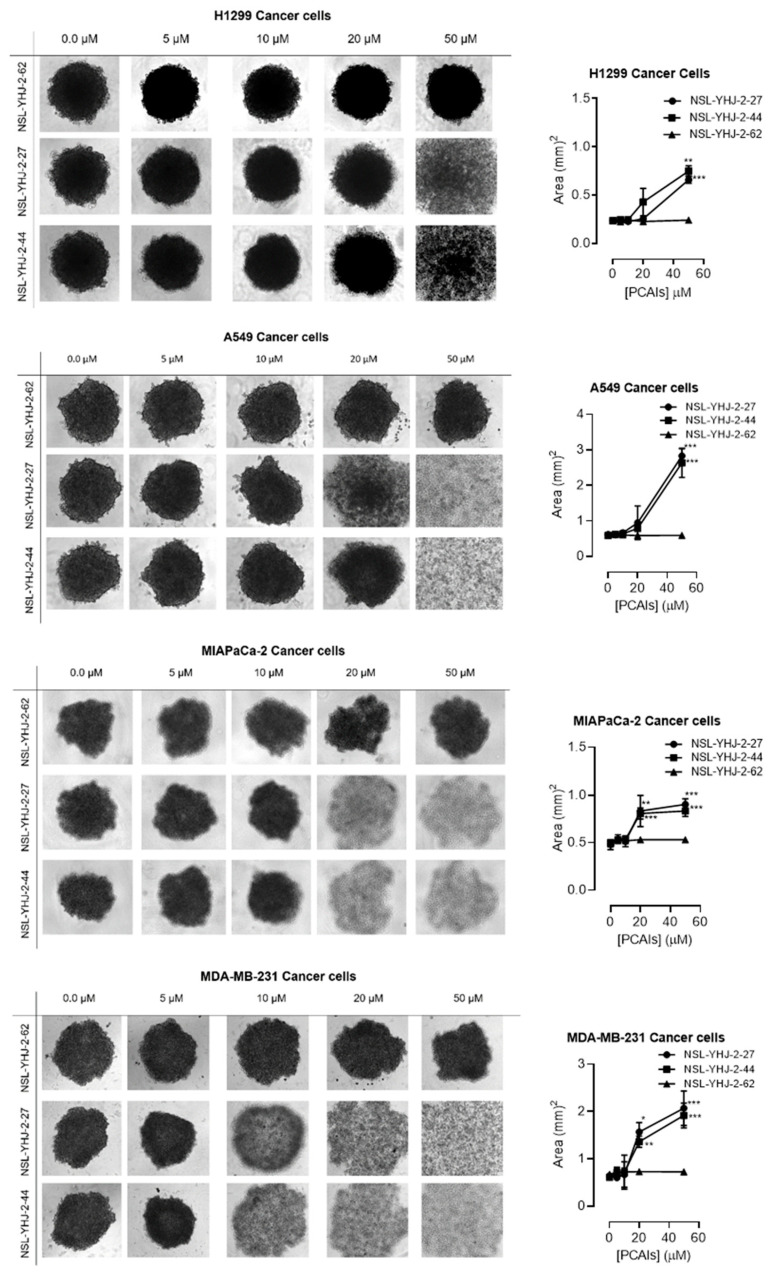
PCAIs induce the degeneration of 3D spheroid cultures. Cells were seeded in Nunclon Sphera 96-well, U-shaped-bottoms microplates and allowed to grow and form compact spheroids overnight. They were then treated with the indicated concentrations of the respective PCAIs for 48 h. The effects of the PCAIs were captured using the Nikon Eclipse Ti 100 inverted microscope (4× magnification) with Nikon DS Qi2 camera. At higher concentrations of PCAIs, the spheroids disintegrate. The effect of the PCAIs on spheroid cultures was quantified by measuring the areas of the spheroid bodies using the NIS-Element software. The results are the mean ± SEM, *n* = 3. Statistical significance (* *p* < 0.05, ** *p* < 0.01 and *** *p* < 0.001) was determined using one-way ANOVA with post hoc Dunnett’s tests.

In order to better understand how effectively the PCAIs may perform against in vivo tumors that are three-dimensional in nature, 3D spheroids were prepared and used to test the effectiveness of the PCAIs [22]. It was observed that the spheroid viabilities were suppressed upon treatment with low micromolar concentrations of the most potent of the PCAIs. As shown in Figure 3, NSL-YHJ-2-27 and NSL-YHJ-2-44 induced the degeneration of spheroids preformed from either A549, NCI-H1299, MIAPaCa-2, or MDA-MB-231cells. Treatment with 50 µM of NSL-YHJ-2-27 and NSL-YHJ-2-44 resulted in over two-fold increases in the area of the spheroid body relative to controls in all the cell lines tested. Interestingly, compound NSL-YHJ-2-62, which lacks the S-farnesyl group, had no effect on the spheroids. As would be expected, the induction of spheroid degeneration and cell death occurred at relatively higher concentrations than the 2D monolayer cultures.

### 3.4. PCAIs Induce Cell Death by Apoptosis

**Figure 4:**
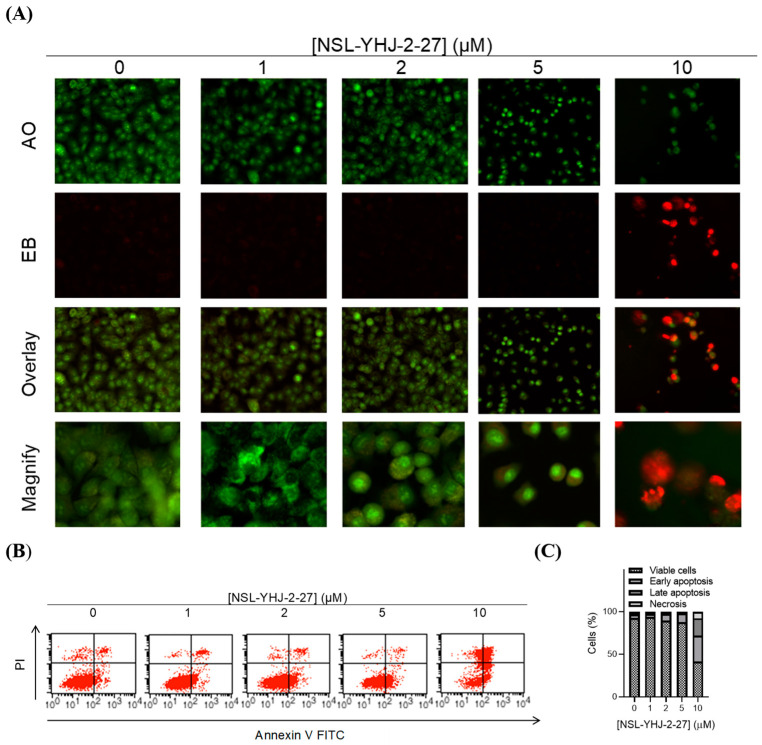
PCAIs induce apoptosis of A549 cells. A549 cells were treated with NSL-YHJ-2-27 (0–10 µM) for 24 h. (**A**) Cells stained with AO/EB (100 mg/mL). Uniformly green color depicts live cells, while bright-green and red depict early and late apoptosis, respectively. (**B**) Cells stained with annexin V and propidium iodide and analyzed using flow cytometry as described in the methods. Live cells remain in the bottom left quadrant. Early and late apoptotic cells appear in the bottom right and top right quadrants, respectively. The top left quadrant is representative of cells undergoing necrosis. (**C**) Bar graphs showing the distribution of cells as determined by flow cytometry analysis.

**Figure 5:**
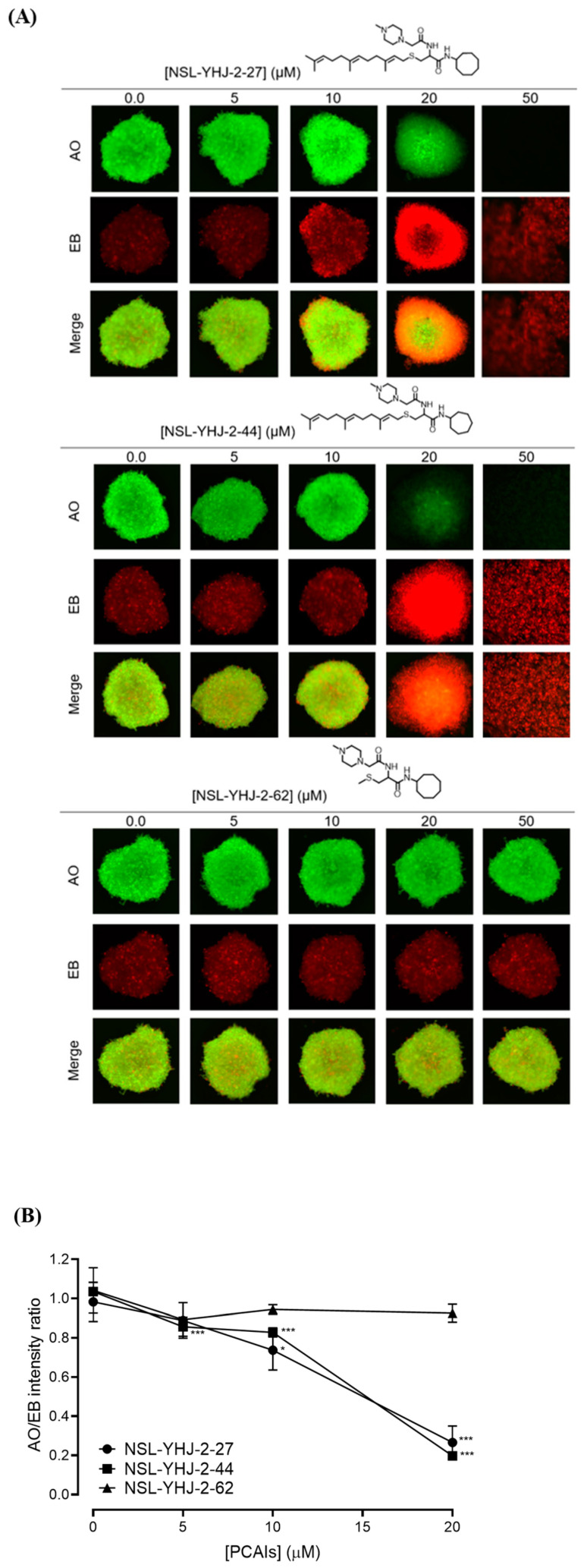
PCAIs induce concentration-dependent cell death in A549 spheroids. (**A**) Cells were seeded in Nunclon Sphera 96-well, U-shaped-bottom microplates and allowed to grow and form compact spheroids overnight. They were then treated with the indicated concentrations of the respective PCAIs for 48 h. The spheroids were stained with AO/EO (5 μg/mL), and the fluorescent images were taken at 4× magnification using the Nikon Ti Eclipse microscope. (**B**) Graph showing concentration-dependent decreases in mean AO/EB intensity ratios (*n* = 3) indicating the viable to non-viable cells ratios in the spheroids. Statistical significance (* *p* < 0.05 and *** *p* < 0.001) was established using one-way ANOVA followed by Dunnett posttest comparisons.

We investigated the mode of cell death induced by the new PCAIs in A549 cells using the AO/EB staining method. As shown in Figure 4A, treatment of A549 cells with NSL-YHJ-2-27 resulted in bright-green fragmented nuclei after 48 h treatment with 2–10 µM of NSL-YHJ-2-27. Cells treated with high concentrations of NSL-YHJ-2-27 are orange-to-red with highly condensed nuclei. To quantify the cells at various stages of apoptosis, we used the Annexin V/PI staining and flow cytometry analysis method. As shown in Figure 4B, exposure to varying concentrations of NSL-YHJ-2-27 (1–10 µM) resulted in higher populations of early and late apoptotic cells (3.0 ± 0.3% to 13.3 ± 7.0%) compared to untreated controls (2.0 ± 0.1%). We also observed a concentration-dependent induction of apoptosis when cells were exposed to the PCAIs. Less than 1% of the cell population was necrotic following treatment with 1, 2, and 5 µM of NSL-YHJ-2-27 (Figure 4C). Moreover, the PCAIs induced concentration-dependent cell death in the A549 spheroids, as indicated by the increase in EB staining (Figure 5). By comparison, the untreated control spheroids were predominantly green due to the exclusion of EB from the cells with intact membranes. The increasing intensity ratios of AO/EB with increasing PCAIs concentrations are an indication of the increasing proportion of dead over live cells in the spheroids since.

### 3.5. PCAIs Activate the MAPK Pathway

**Figure 6:**
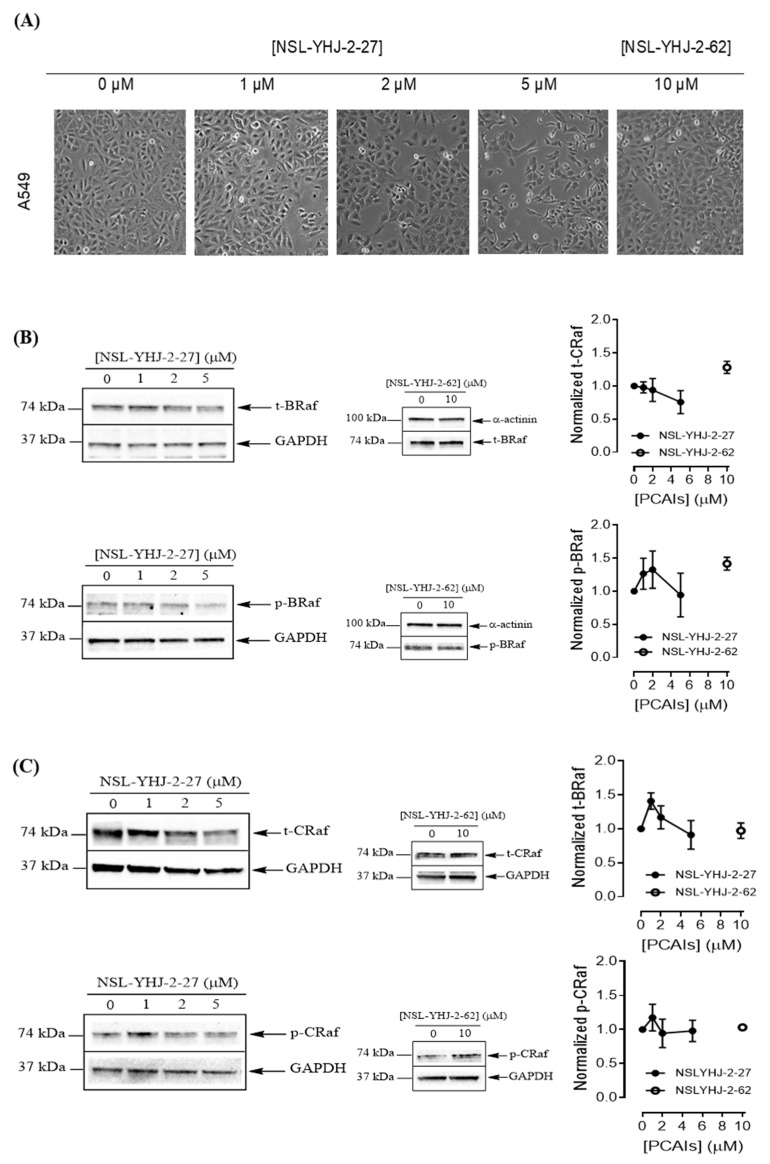

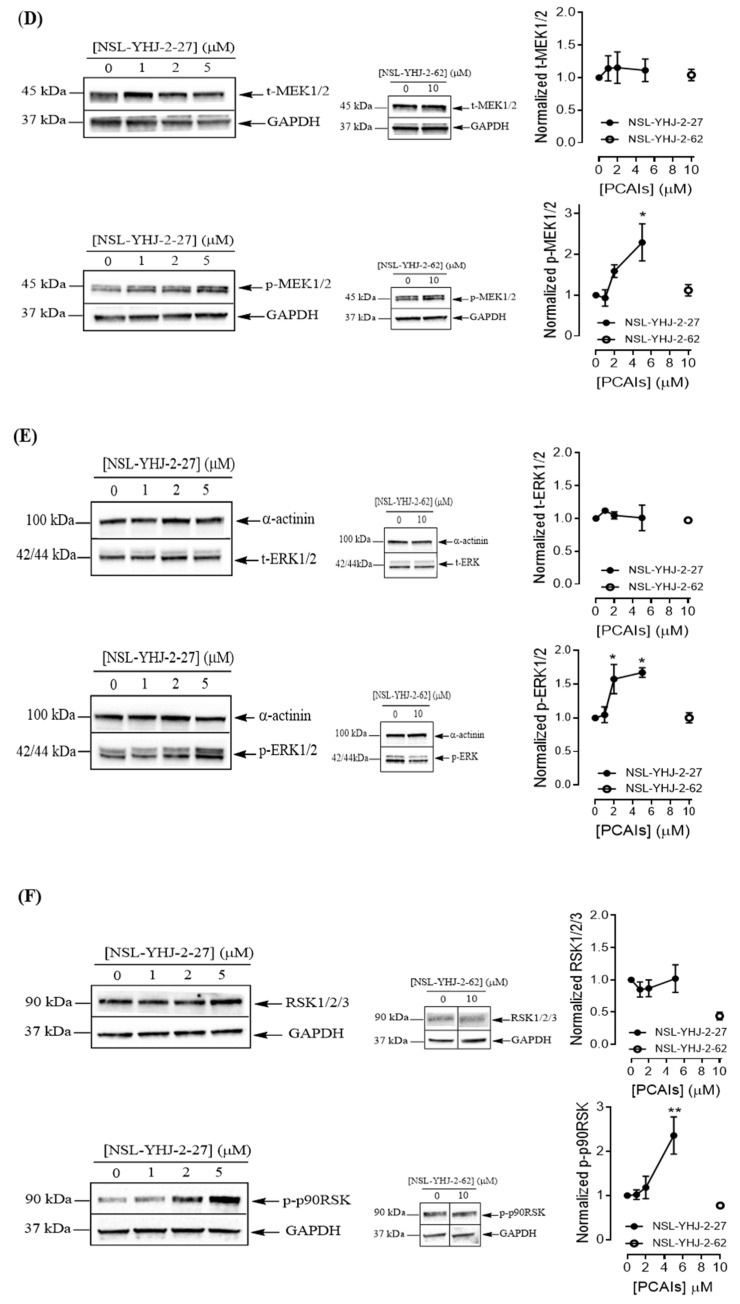
PCAIs activate the MAPK pathway in A549 lung cancer cells. A549 cells were treated for 48 h with 0−5 μM of NSL-YHJ-2-27 or 10 μM of NSL-YHJ-2-62. These were then lysed and subjected to western blot analysis for total and phosphorylated levels of MAPK pathway proteins. (**A**) Shows treated cells captured using the Nikon Eclipse Ti 100 inverted microscope at 10× magnification just before lysis for western blotting analysis. (B-F) Western blot images and densitometry plots of bands following quantification using Image Lab Software normalized against GAPDH or α-Actinin. The samples were analyzed for total (**B**) BRaf (t-BRaf) and phosphorylated BRaf (p-BRaf); (**C**) total CRaf (t-CRaf) and phosphorylated CRaf (p-CRaf); (**D**) total MEK1/2 (t-MEK1/2) and phosphorylated MEK1/2 (p-MEK1/2); (**E**) total ERK1/2 (t-ERK1/2) and phosphorylated ERK1/2 (p-ERK1/2); and (**F**) total p90RSK (t-p90RSK) and phosphorylated p90RSK (p-p90RSK). Data are representative of three independent experiments. Statistical significance (* *p* < 0.05 and ** *p* < 0.01) was determined by 1-way ANOVA with post hoc Dunnett’s test. Original blots see Figure S1.

To understand the anticancer mechanisms of the PCAIs, we explored the effects of the newer PCAIs on the MAPK pathway using lung cancer A549 cells (Figure 6A). When the cells were treated with NSL-YHJ-2-27 for 48 h, no significant difference was observed in the levels of total or phosphorylated B- and C-Raf proteins (Figure 6B,C). However, it was observed that the PCAIs immensely stimulated the MAPK pathway downstream of the Raf proteins in a concentration-dependent manner following treatment with 1, 2, and 5 μM of NSL-YHJ-2-27. The phosphorylation of MEK1/2, ERK1/2, and p90RSK significantly increased by 84%, 59%, and 160%, respectively, relative to controls following treatment for 48 h with 5 µM of NSL-YHJ-2-27 (Figure 6D–F). Interestingly, a non-farnesylated analog, NSL-YHJ-2-62, did not elicit any such effects on the MAPK pathway (Figure 6D–F).

## 4. Discussion

**Figure 7:**
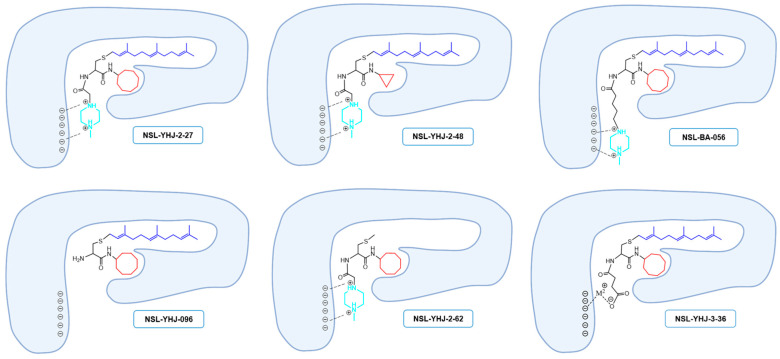
Structure–activity-relationship-based depiction of possible PCAIs interactions with potential targets. Schematic diagrams showing the possible ionic and hydrophobic interactions of the analogs with the target are based on the relative effects of the analogs against cancer cell viabilities. The most potent analogs (NSL-YHJ-2-27 and NSL-BS-056) are believed to have the most cooperative binding contacts for higher affinities and potencies. Although other factors such as the degree of cell entry may be in play, the surprising effectiveness of NSL-YHJ-3-36 suggests possible contribution of salt bridge to its effectiveness.

Previous studies on the PCAIs revealed their effectiveness against such cancer phenomena as cell viability, angiogenesis, cell migration and invasion, and cytoskeletal organization [19,20,21,23,24,25,26]. The present study reports on the synthetic optimization for increased potency and bioavailability as well as the determination of the anticancer molecular mechanisms. Contrary to our previous expectations that an ionizable group tethered to the ∝-amino group of cysteine will serve mainly to enhance the aqueous solubility of the PCAIs [19], the present study reveals an additional desirable effect on potency since a PCAIs analog lacking a tethered ionizable group (NSL-YHJ-096) was up to 10 times less potent against the cell lines. The presence of a tether between the cysteinyl amino group and the ionizable moieties appears to be more crucial for potency than its actual length since tether lengths ranging from two to six atoms yielded moderately variable but very effective PCAIs. The failure of NSL-YHJ-2-84 to show any effect on cell viability signifies the importance of the ionizable groups at the end of the tethers. In addition to improving the aqueous solubilities of the PCAIs, the ionizable groups appear to contribute to affinity as well as a possible entry into cells. Positively charged groups are more likely to bind to membranes that are negatively charged, thereby facilitating entry into the cells. This benefit would be lacking in neutral and negatively charged compounds. PCAIs with positively charged tethered methylpiperizine moderately varied in potency depending on the length of the tether. This may reflect more than just contributions to solubilities and cell entry but also interactions with intracellular targets. The tethered positive charges of the PCAIs may somewhat mimic the positive charges of the polybasic regions of G-proteins such as KRAS. If the polybasic region of KRAS plays a role in the protein-protein interactions in addition to the well documented role played by the farnesyl group, the positive charges of the ionized piperizinyl moiety may play an analogous role when the PCAIs bind to the same sites to uncouple proteins such as KRAS from their polyisoprenyl-dependent interactions with other proteins. This is corroborated somewhat by the lack of effect in control compounds without the polyisoprene that showed no effect even at 50 µM. Similarly, low potencies against cell viability displayed by the neutral or the negatively charged analogs confirm the requirement for a positively charged side chain for potency. That the negatively charged analog will have activity was somewhat surprising but understandable since a salt bridge involving metal ions such as Ca^2+^ would still promote binding interactions (Figure 7).

Although more cell lines without the KRAS mutation need to be tested to fully understand the targeted nature of the PCAIs on cell viability of KRAS-mutant cells, the observation that the cell lines with the mutant KRAS driver are relatively more susceptible to the PCAIs suggests that the PCAIs are impacting a KRAS-mediated process. Of the RAS isoforms, KRAS is the only one in which a single C-terminal cysteine is modified [27]. This implies that cancers driven by KRAS may be more susceptible to the PCAIs as the essential polyisoprenyl-driven functional interactions may be more easily uncoupled than would be the case for the other RAS proteins that are farnesylated and palmitoylated [27]. It has indeed been reported that RAS proteins modified only through farnesylation can dissociate more rapidly from membranes than those anchored through farnesylation and palmitoylation [28,29]. This may help explain the difference between the effects of the PCAIs on cell lines driven by KRAS mutants compared to those driven by other mutant RAS isoforms.

PCAIs suppression of cell viability was expected to be associated with the suppression of the MAPK pathway since RAS activation is reported to activate RAF proteins that constitute the first members of the pathway [2,30]. It was thus surprising to see instead increased MEK1/2, ERK1/2, and p90RSK phosphorylation, which are all synonymous with MAPK pathway activation. Although MAPK stimulation has mostly been associated with cell survival and proliferation, our findings are consistent with various studies showing that ERK activation can also mediate apoptosis depending on the intensity and duration of the stimulation as well as the type of cell line and stimuli [31,32,33,34]. The prolonged ERK1/2 activation was reported to be pro-apoptotic [34,35]. Although the mechanism of this ERK-mediated apoptosis is not clear, activated ERK was reported to negatively regulate B-Raf and C-Raf via feedback phosphorylation [36]. Moreover, the observed increase in p90RSK activation after the PCAIs treatment may be due to increased phosphorylation in RSK3 and RSK4 isoforms that have tumor suppressor functions [6,37,38]. While RSK1 and RSK2 play roles in cell growth and proliferation, RSK3 and RSK4 have been reported to promote cell cycle arrest and apoptosis [6,37,38]. Another key ERK substrate with a large footprint in carcinogenesis due to its widespread involvement in the expression of numerous genes is c-Myc. MEK/ERK inhibition results in c-Myc dephosphorylation and apoptosis [39]. The fact that ERK activation upon treatment with the PCAIs resulted in apoptosis suggests that c-Myc may not be affected.

How the PCAIs elicit the phosphorylation of these proteins is not yet clear. RAF interactions with RAS have been reported to involve two regions of RAF: the RAS-binding domain (RBD) and the cysteine-rich domain (CRD) [40]. The farnesylated cysteine plays a significant role in the formation of RAS-RAF complexes through interactions with a hydrophobic surface on CRD [41]. Interactions of the PCAIs with such surfaces may perturb the RAS-RAF interactions. Rather than activate the MAPK pathway, the PCAIs would be expected to suppress RAF activity and consequently the chain of events downstream as it is inconceivable that the PCAIs would directly and adequately assume the role of RAS. Monomeric G-proteins are known to interact with chaperone proteins in a polyisoprenylation-dependent manner [42,43,44,45]. For example, Galectin 8 isoforms have been shown to bind to farnesylated but not to unfarnesylated KRAS [46]. It is possible that the PCAIs may dislodge the sequestered pools of G-proteins from the chaperones, thereby increasing the pool of free KRAS to interact with and activate RAF.

## 5. Conclusions

The effectiveness of the current batch of novel PCAIs demonstrates the potential for their continuous development into cancer therapies. That the PCAIs are effective against cancer cells with and without RAS mutations bodes well for their consideration for treating cancers with broader genetic variations. These effects on a broader range of cancer cell lines, though appearing as not being specific, rather reflect the significant roles of the various G-proteins in various pathways and by extension various aspects of cancer progression. A potential benefit would be wider applicability with relatively fewer escape mechanisms that result in resistance. The results demonstrate the potential for design improvements to further optimize potency and drug properties. Although the MAPK pathway appears to be strongly affected, mechanisms involving other pathways and monomeric G-proteins other than KRAS are likely to be involved and are the subject for future investigations.

## Figures and Tables

**Table 1:** Structure activity relationships of the PCAIs. The physicochemical properties and inhibition of cancer cell viabilities by the PCAIs. The EC_50_ values were obtained from concentration-response curves following 48 h treatment with the indicated PCAIs. Various structures have been color-coded for easier comparisons.

Compound	Structure	Log P	MW	48hEC_50_ (µM)			
				NCI-H1299	A549	MDA-MB-231	MIAPaCa-2
NSL-YHJ-096	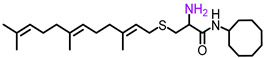	5.59	434.72	50	45	22	22
NSL-YHJ-2-62	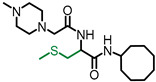	0.8	384.58	>50	>50	>50	>50
NSL-YHJ-2-56	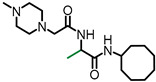	0.58	338.49	>50	>50	>50	>50
NSL-YHJ-31	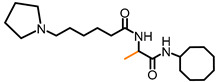	2.54	365.56	>50	>50	>50	>50
NSL-BA-055	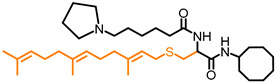	6.91	601.97	4.5	5.6 ^a^	2.6	2.8
NSL-BA-056	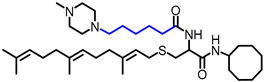	6.77	631.02	12 ^a^	49 ^a^	6.7 ^b^	2.5 ^c^
NSL-YHJ-2-40	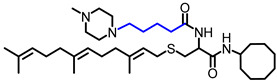	6.35	616.99	5.0	4.4	2.6	5.3
NSL-YHJ-2-37	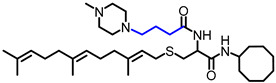	5.9	602.97	5.6	3.4	3.7	6.4
NSL-YHJ-2-35	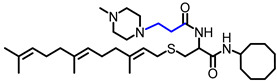	5.51	588.94	6.0	2.9	3.6	5.7
NSL-YHJ-2-27	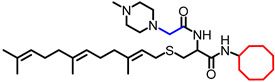	5.09	574.91	5.5	2.2	2.2	2.3
NSL-YHJ-2-44	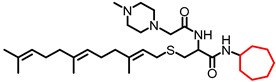	4.67	560.89	6.6	2.3	2.8	2.9
NSL-YHJ-2-45	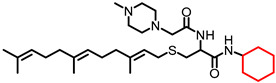	4.26	546.86	7.7	3.9	3.4	4.2
NSL-YHJ-2-46	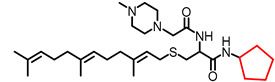	3.84	532.83	8.9	4.7	5.4	5.8
NSL-YHJ-2-47	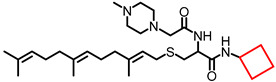	3.42	518.80	9.7	5.5	5.1	5.4
NSL-YHJ-2-48	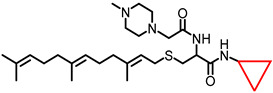	3.01	504.78	14	7.6	6.8	6.5
NSL-YHJ-2-67	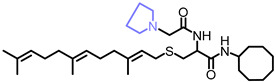	5.6	545.86	16	17	5.2	11
NSL-YHJ-2-27	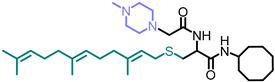	5.09	574.91	5.5	2.2	2.2	2.3
NSL-YHJ-2-89	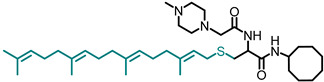	7.11	643.02	5.3	7.0	2.4	4.1
NSL-YHJ-2-84	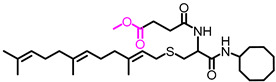	5.45	548.83	>50	>50	>50	>50
NSL-YHJ-3-36	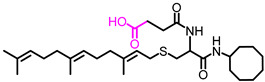	5.19	534.80	15.4	>50	24	23

^a^ [20] ^b^ [21] ^c^ [19].

## Data Availability

Data sharing is not applicable to this article.

## Supplementary Materials

The following are available online at https://www.mdpi.com/article/10.3390/cancers13225757/s1, Supplementary S1: original blots.
