# Supplementary material for: Synthetic Optimization and MAPK Pathway Activation Anticancer Mechanism of Polyisoprenylated Cysteinyl Amide Inhibitors
Source: Cancers (Basel). 2021 Nov 17;13(22):5757. doi: 10.3390/cancers13225757 (PMC8616522; doi:10.3390/cancers13225757)

Supplementary S1:

Figure 6

(B)

Total BRaf-NSL-YHJ-2-27

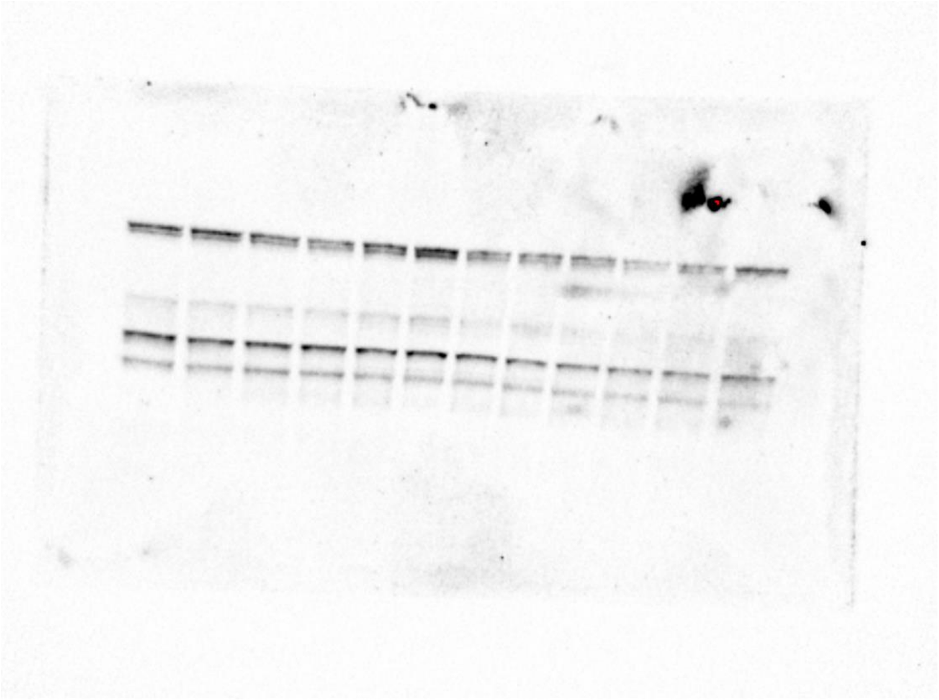

Control

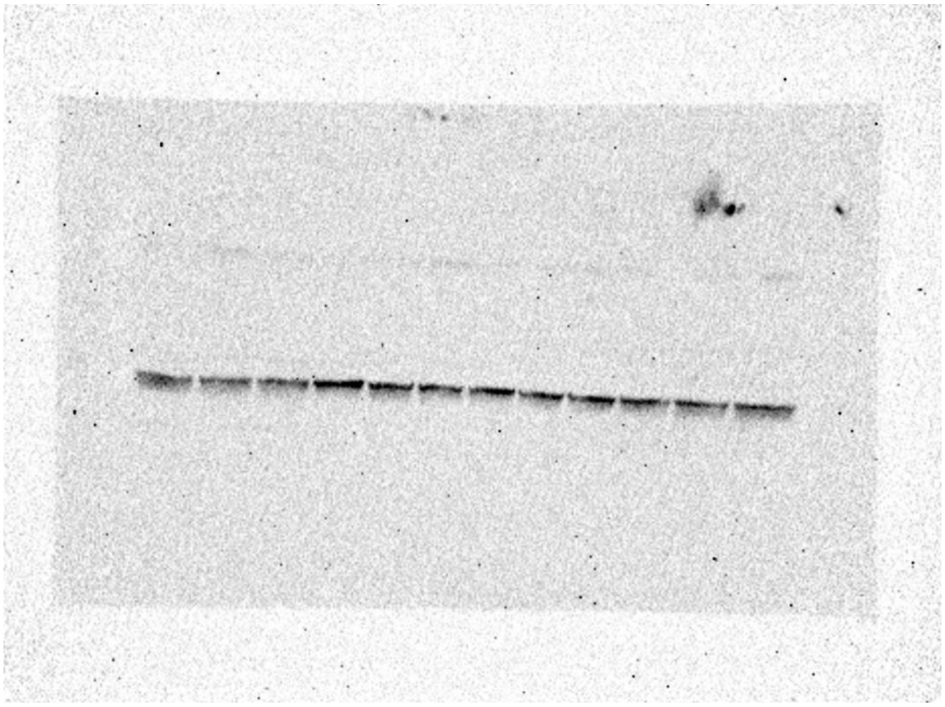

Total BRaf-NSL-YHJ-2-62

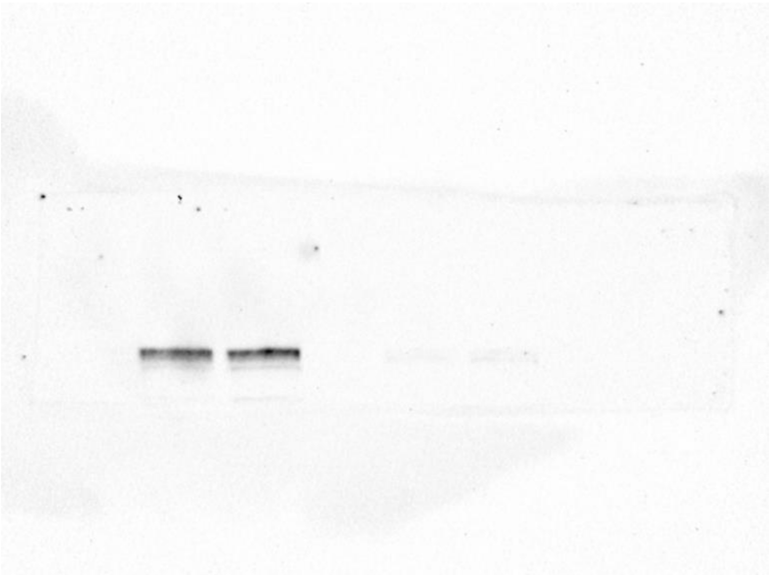

Control

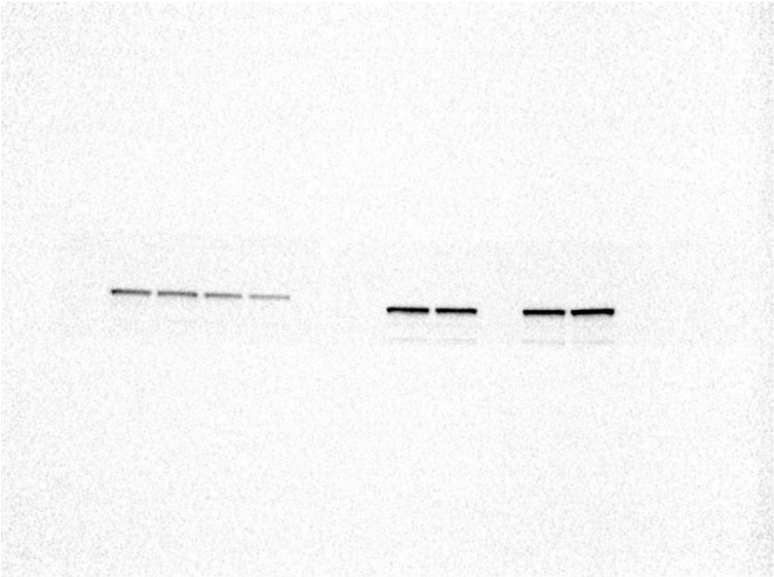

p-BRaf NSL-YHJ-2-27 and NSL-YHJ-2-62

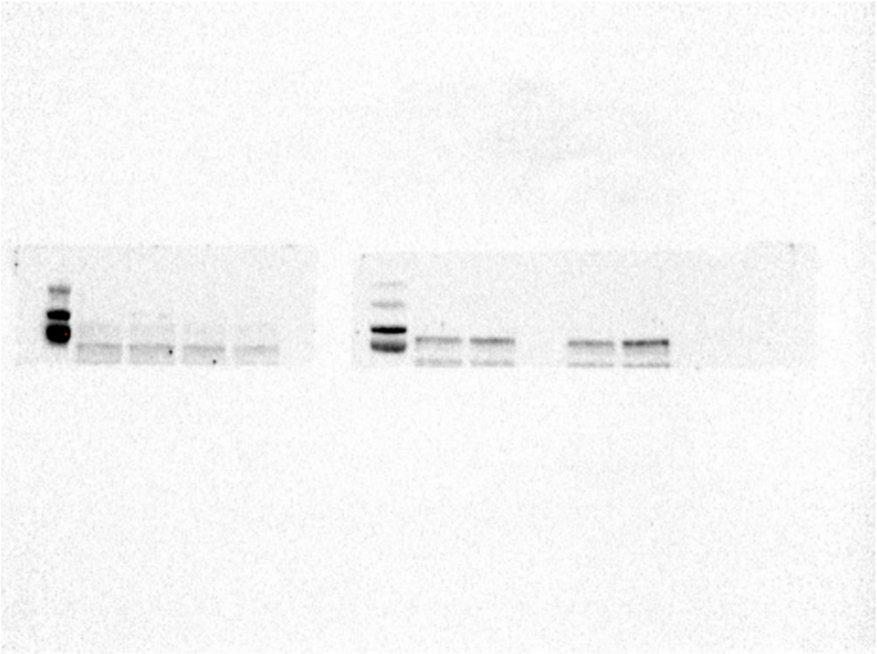

Control

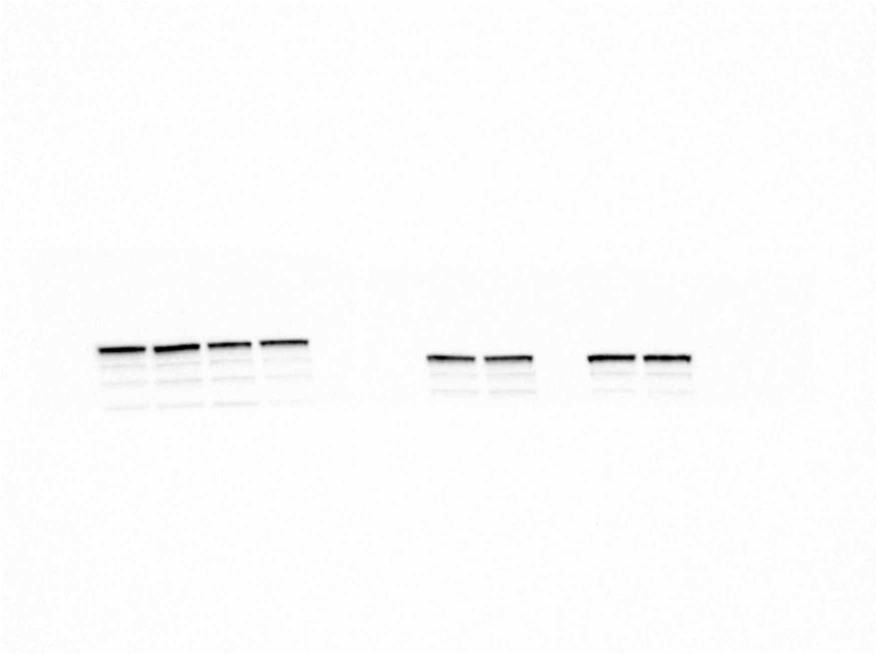

(c)

Total CRaf-NSL-YHJ-2-27

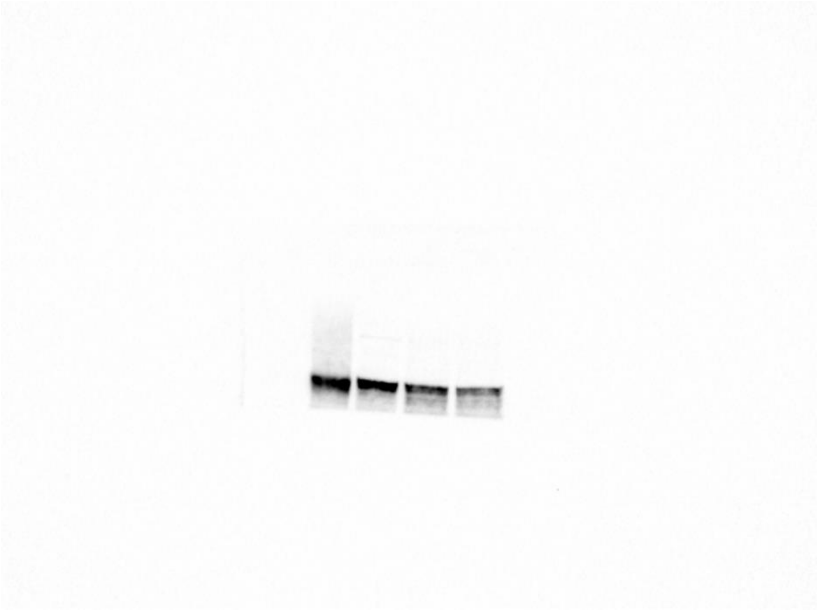

Control

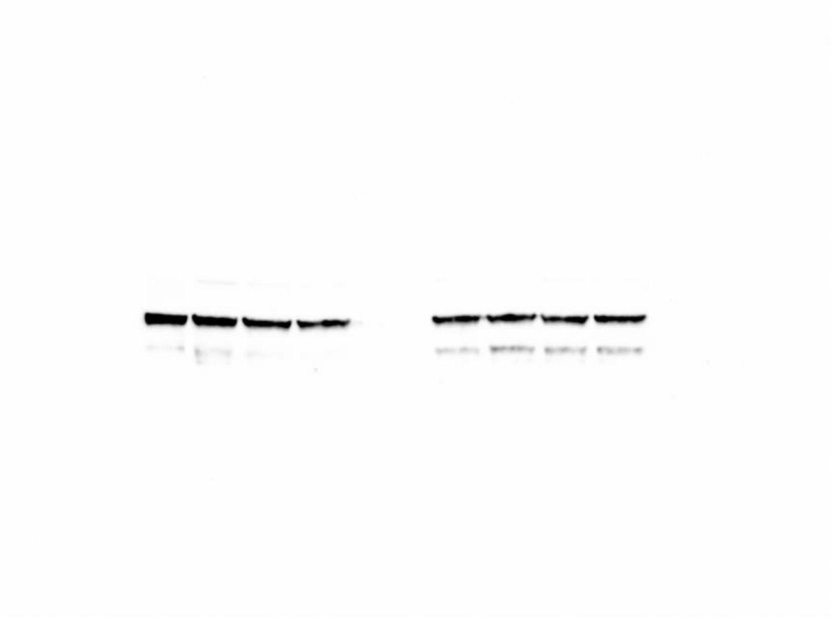

Total CRaf-NSL-YHJ-2-62

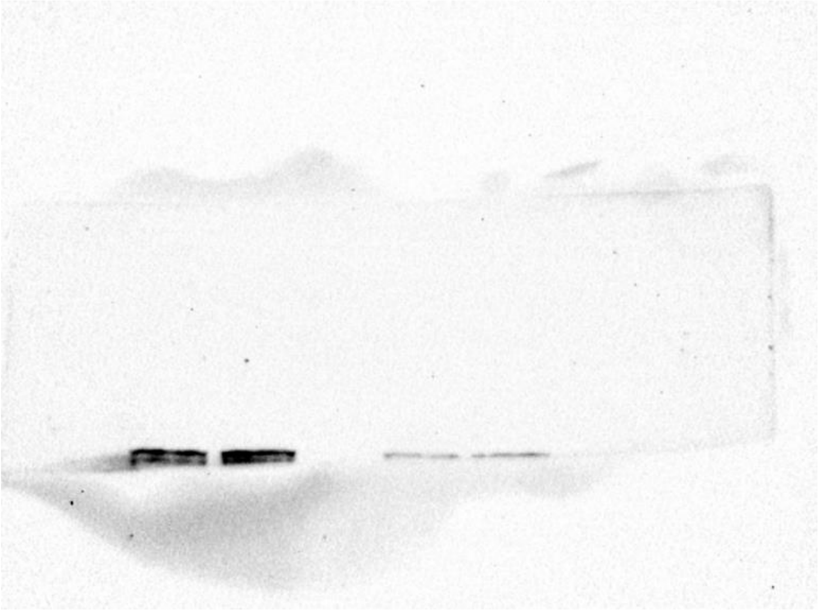

Control

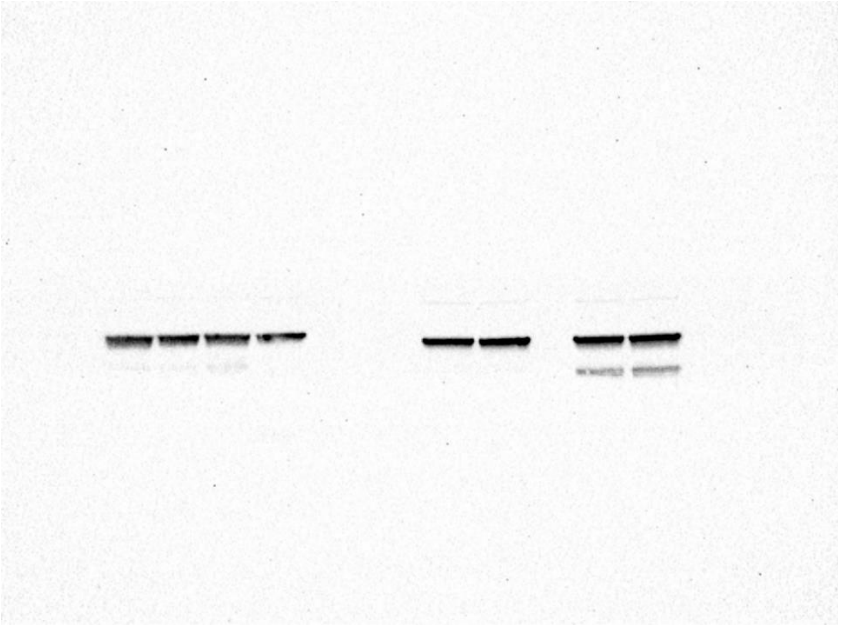

P-CRaf-NSL-YHJ-2-27

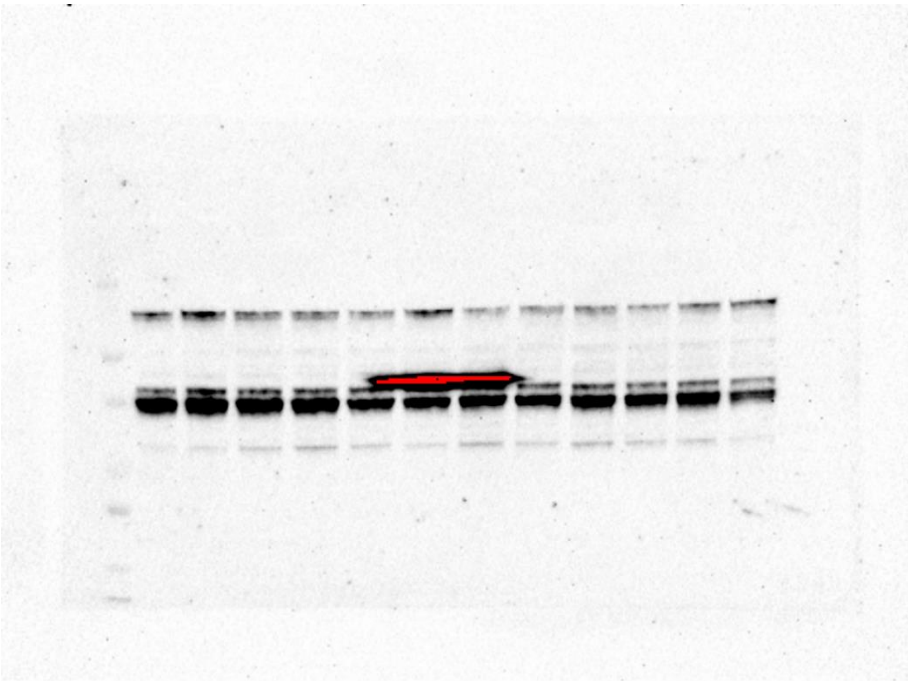

Control

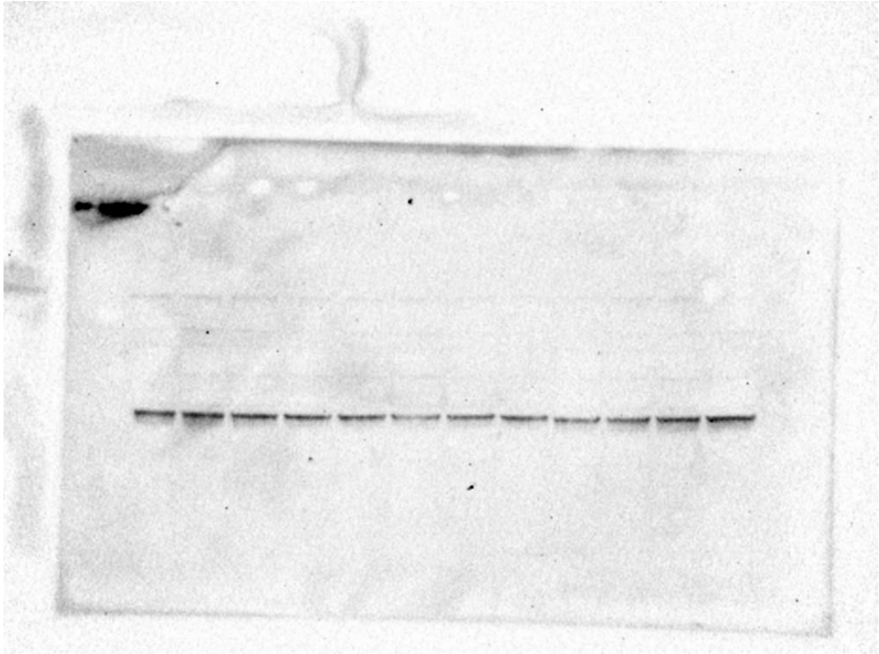

p-CRaf-NSL-YHJ-2-62

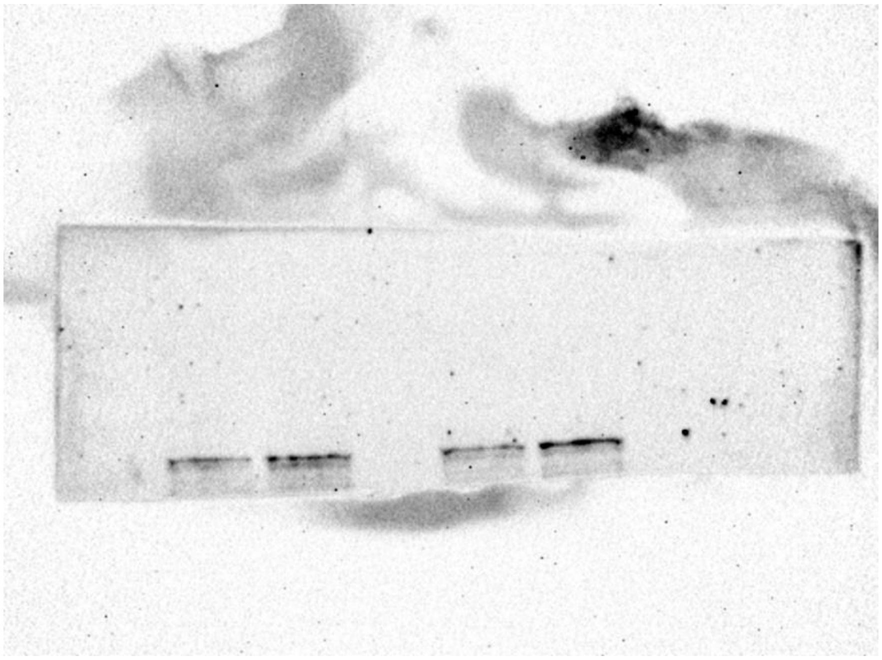

Control

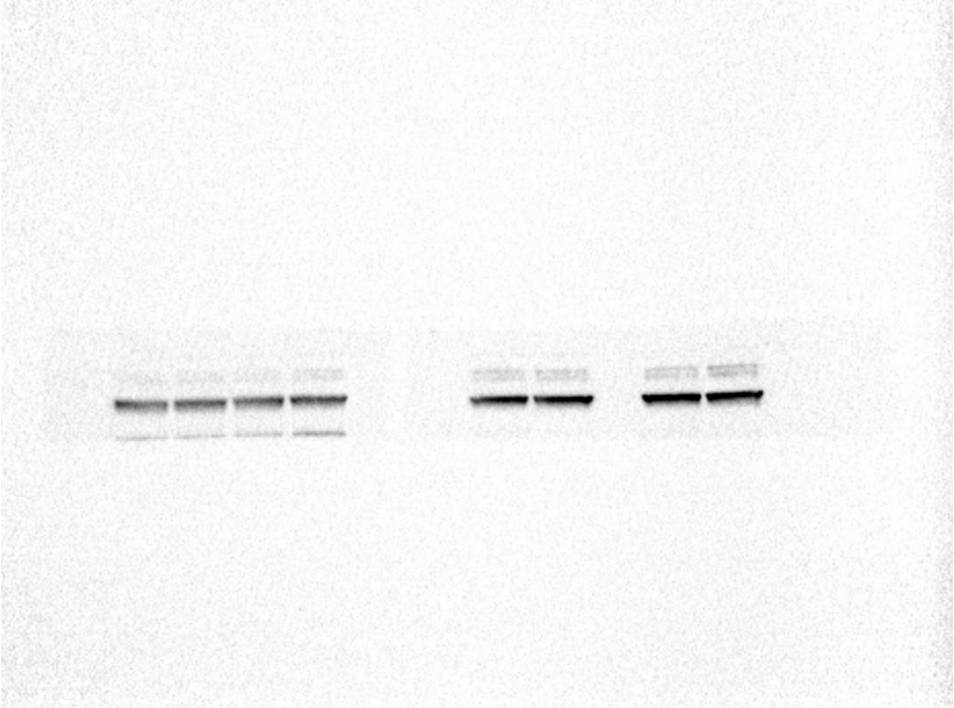

(D)

Total MEK1/2-NSL-YHJ-2-27

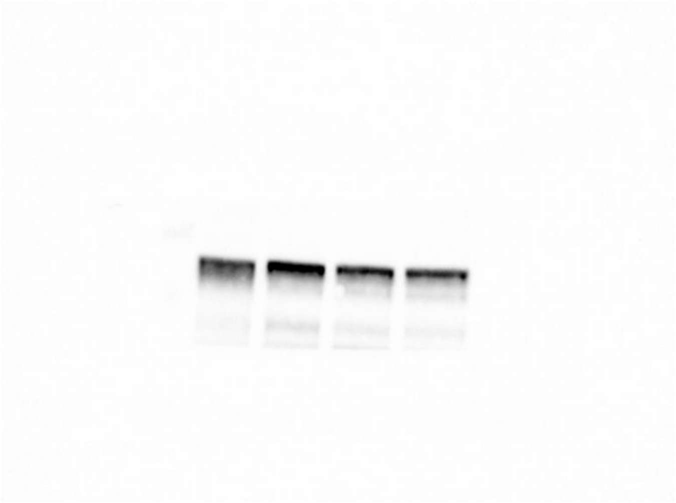

Total BRaf-NSL-YHJ-2-62

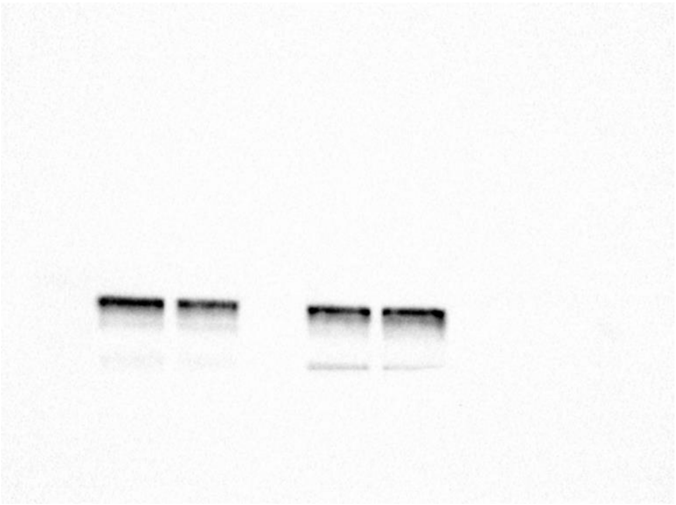

Control

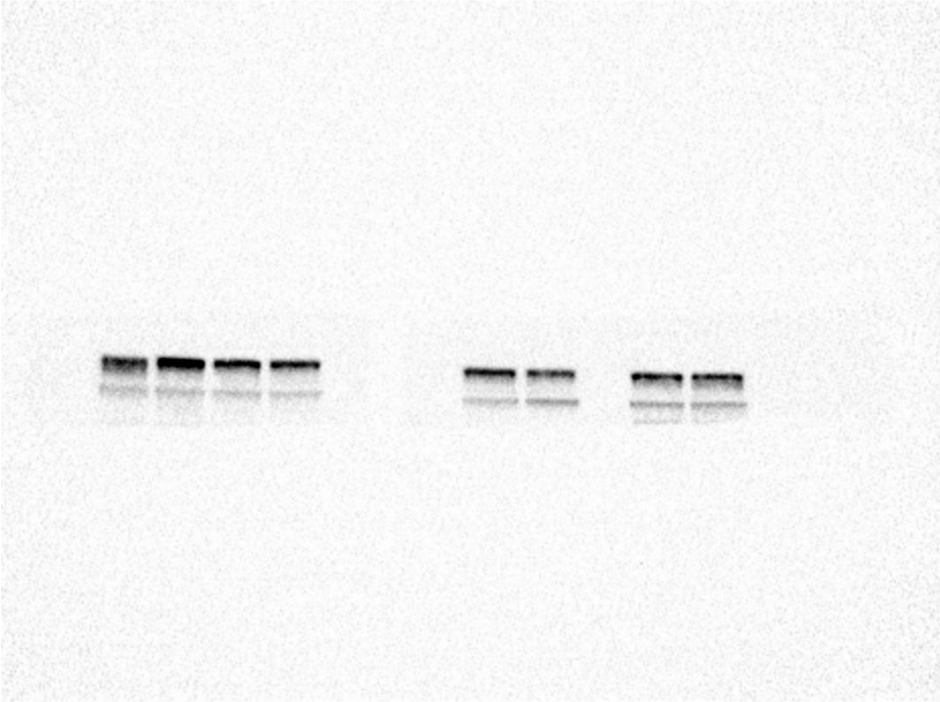

p-MEK1/2-NSL-YHJ-2-27

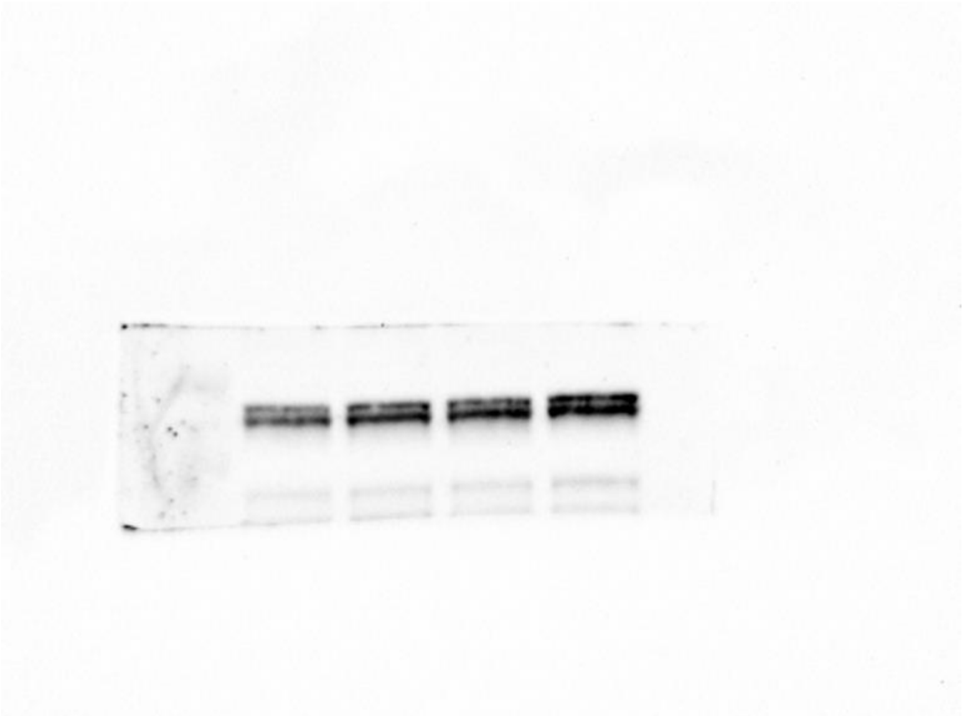

p-MEK1/2-NSL-YHJ-2-62

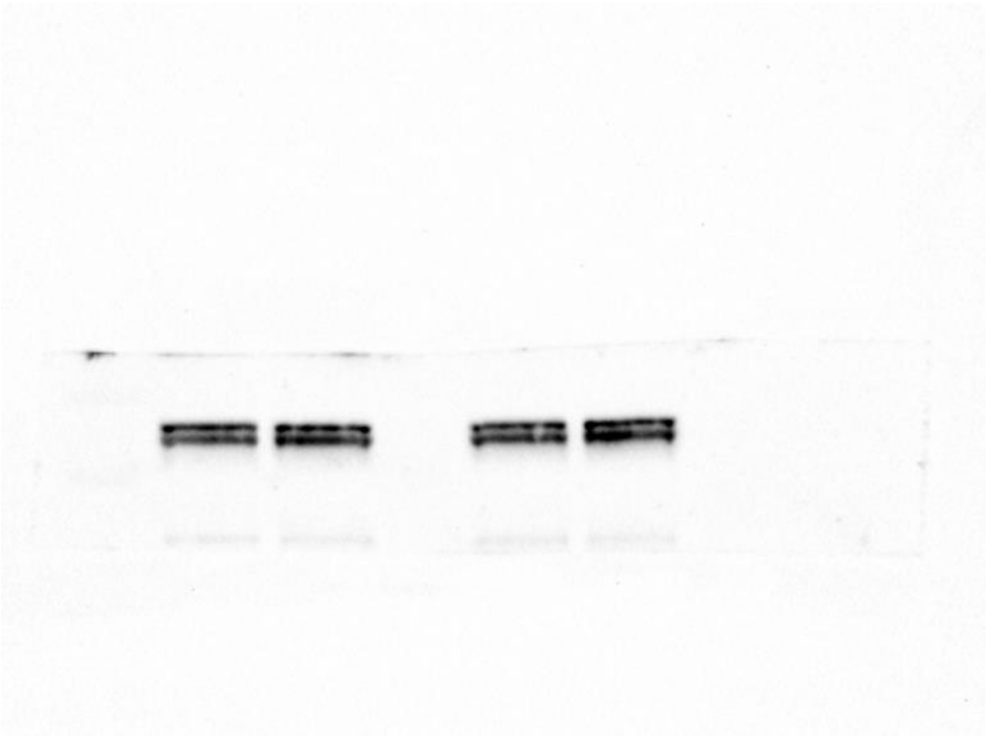

Control

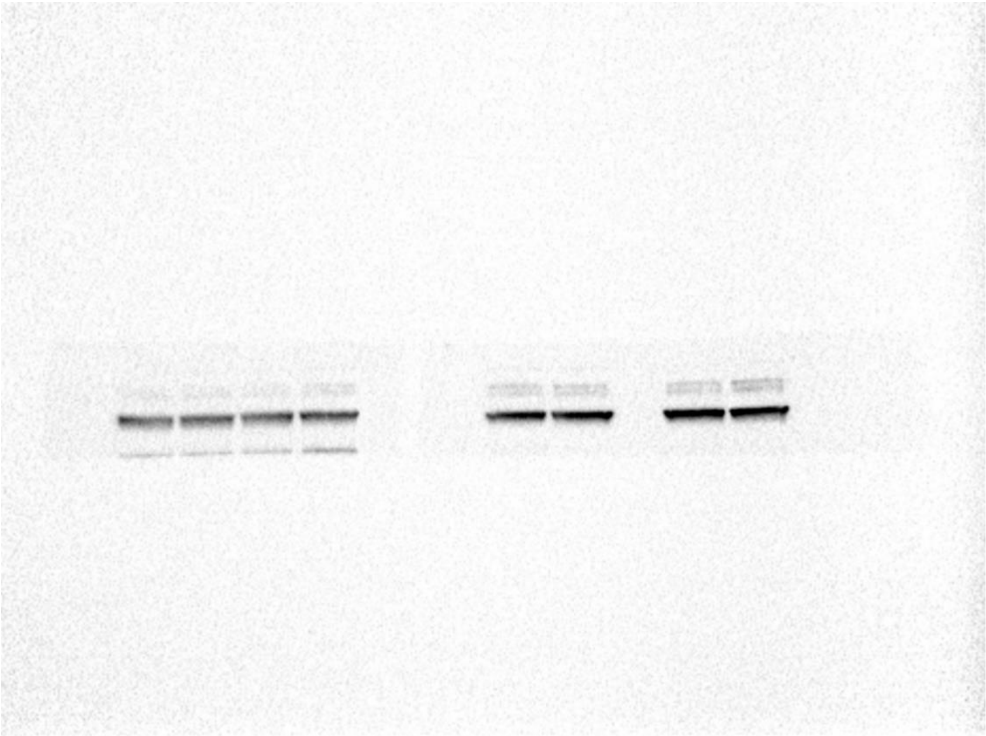

(E)

Total ERK1/2-NSL-YHJ-2-27

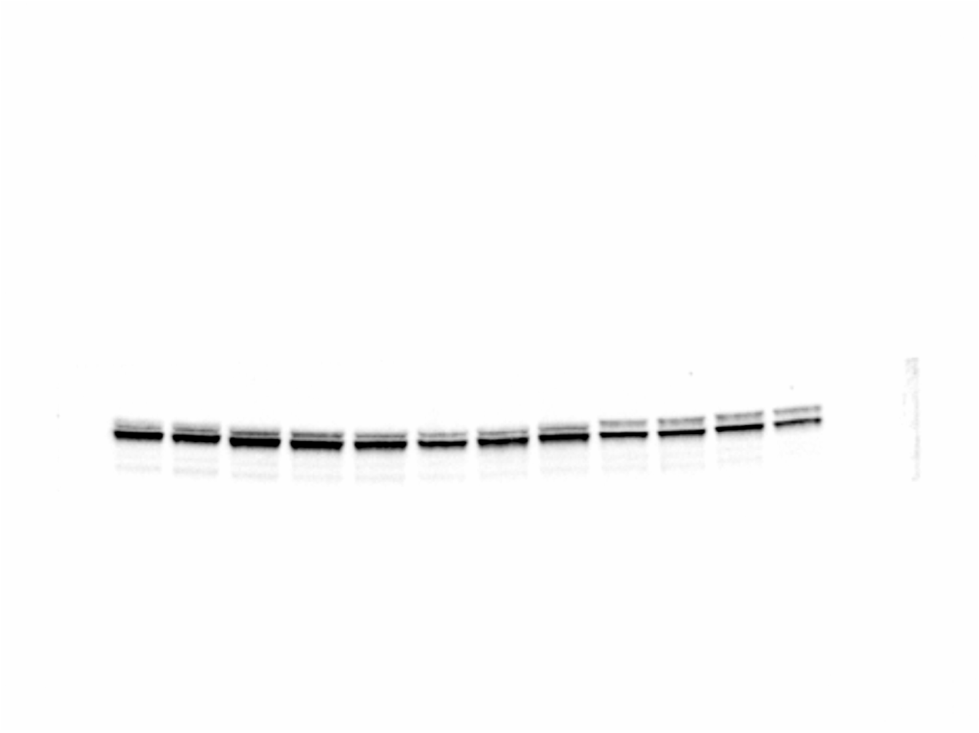

Control

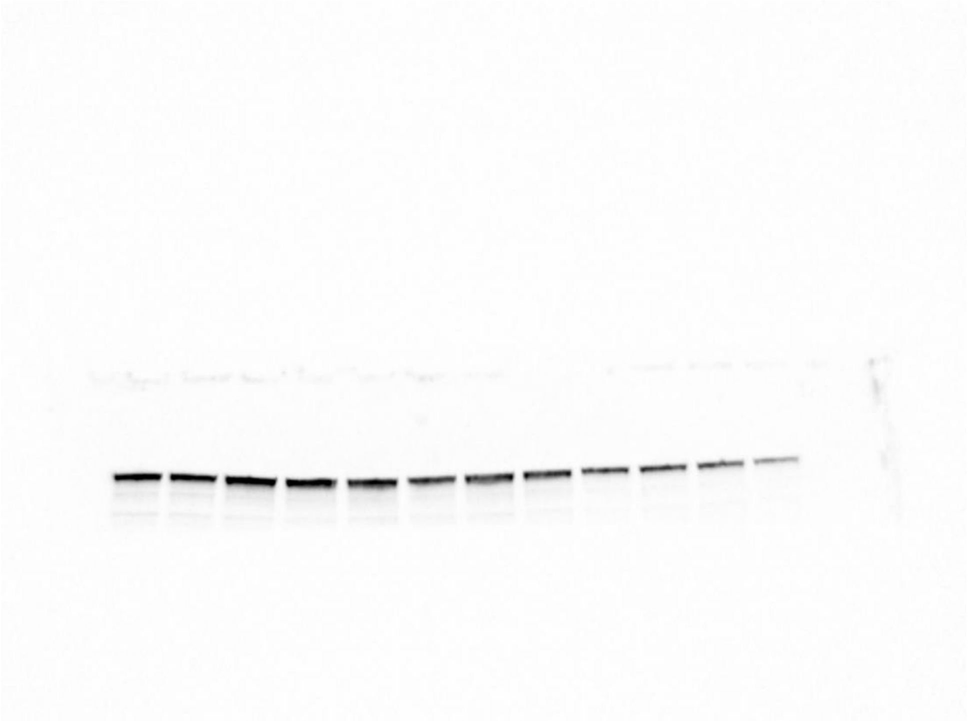

p-ERK1/2-NSL-YHJ-2-27

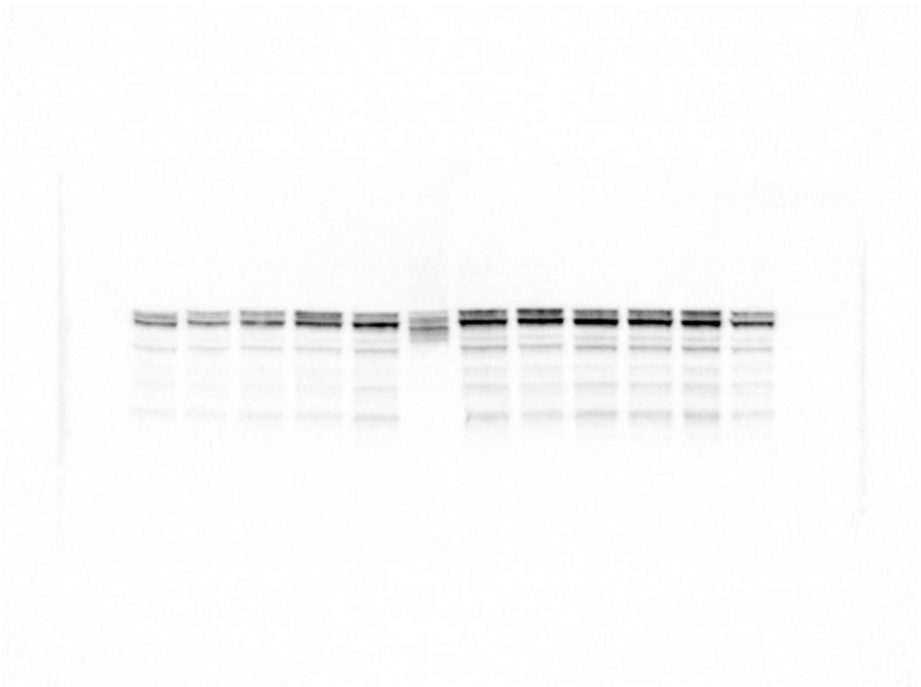

Control

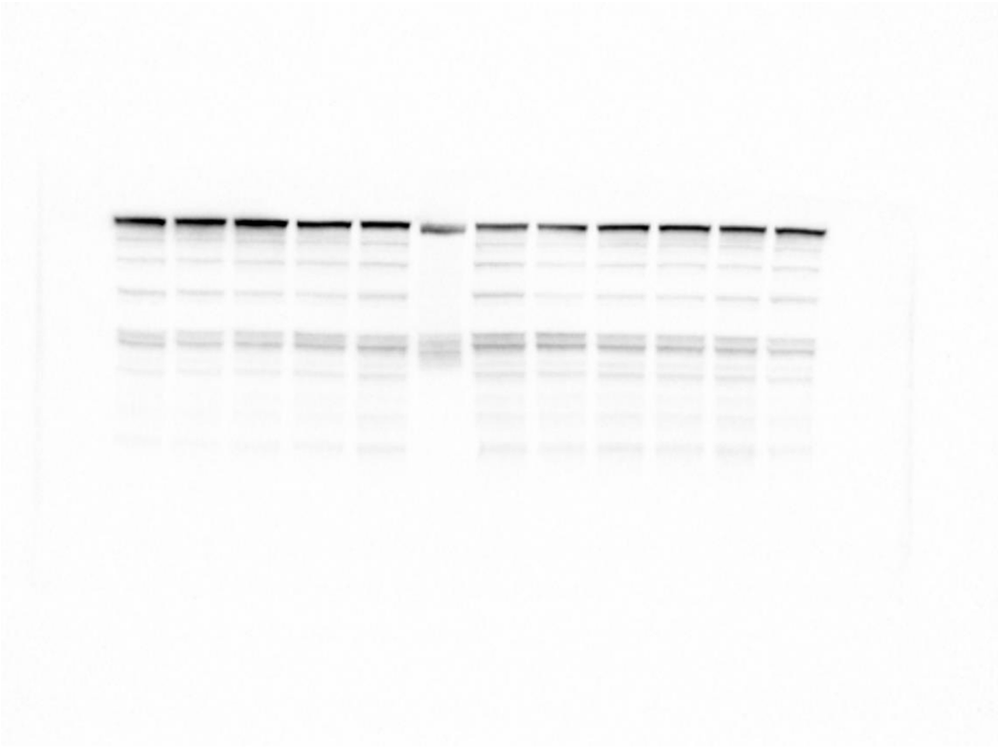

(F)

Total RSK1/2/3 NSL-YHJ-2-27 and NSL-YHJ-2-62

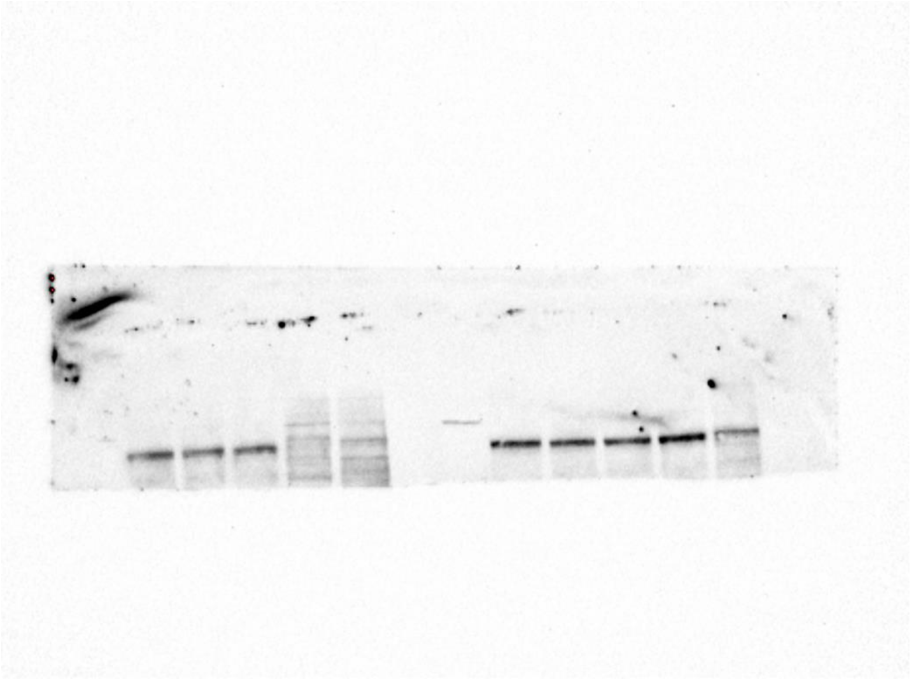

Control

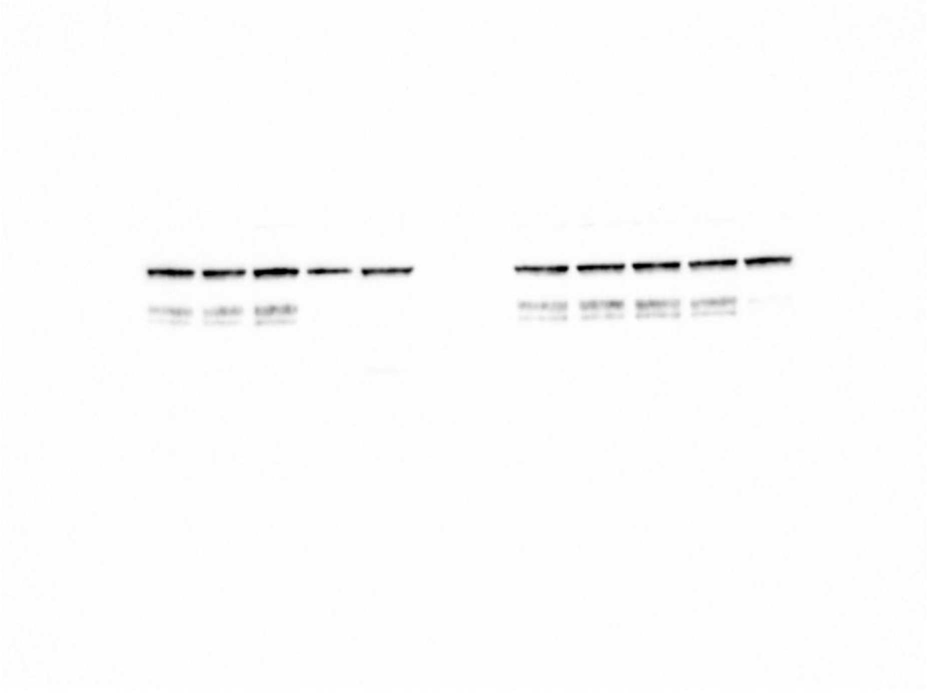

p-p90RSK NSL-YHJ-2-27 and NSL-YHJ-2-62

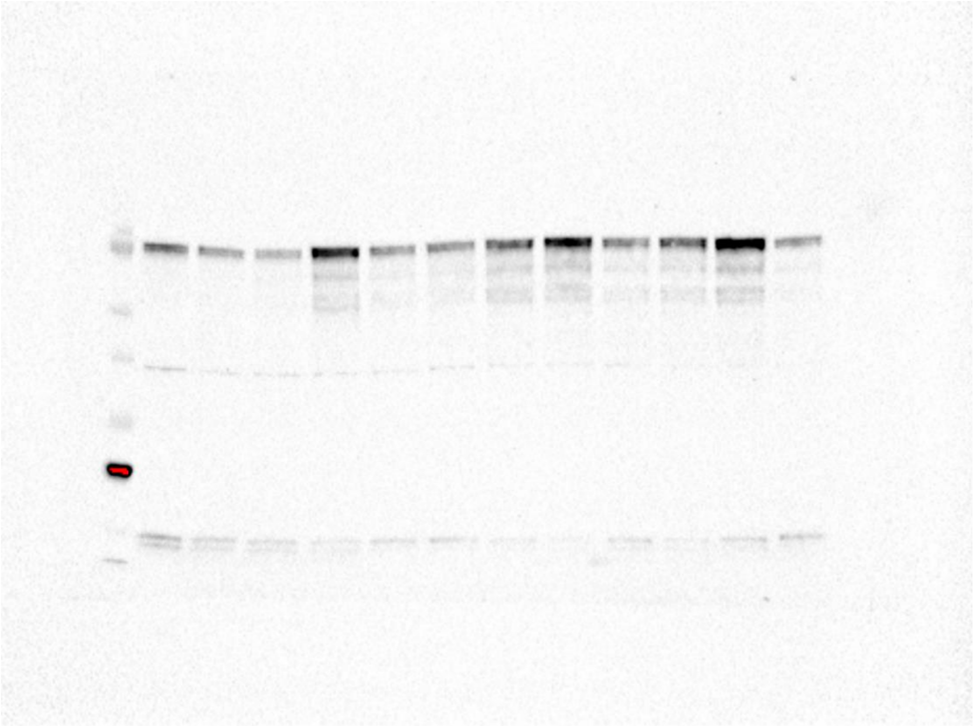

Control

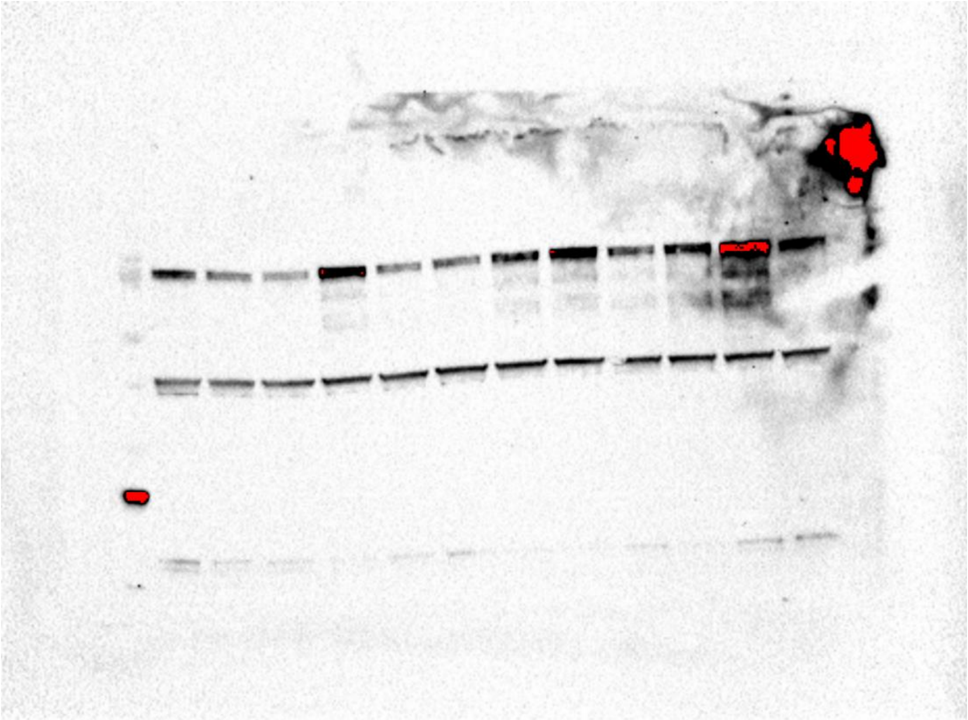

Supplement: Supplementary file 1 [file cancers-13-05757-s001.zip › cancers-1447551-supplementary.pdf]
